# Multi-hazard exposure in public schools of a medium-sized city: outdoor road dust contamination, and indoor Rn risk

**DOI:** 10.1007/s10653-026-03313-6

**Published:** 2026-06-29

**Authors:** Lara Almeida, Fernando Rocha, Alcides Pereira, Cristina Sequeira, Carla Candeias

**Affiliations:** 1 GeoBioTec Research Unit, Department of Geosciences, University of Aveiro., Campus de Santiago, 3810-193 Aveiro, Portugal; 2 Institute D. Luiz, Department of Earth Sciences, University of Coimbra, 3030-299 Coimbra, Portugal

**Keywords:** Outdoor road dust, Indoor Rn, Public schools, Risk assessment, Human health

## Abstract

Road dust is a major carrier of potentially toxic elements (PTEs) in urban environments. This study characterizes road dust samples collected in the vicinity of public schools (n = 17) to assess their physicochemical properties, mineralogy, chemical composition, magnetic susceptibility, particle morphology, contamination indices, and gastric bioaccessibility of some PTEs (< 250 µm fraction). Samples were mainly composed of quartz, with feldspars, Σphyllosilicates, carbonates, and magnetite-maghemite, revealing geogenic and anthropogenic sources, e.g., tire wear, brake abrasion, and resuspended pavement debris. The fraction < 106 µm was enriched in PM_10_, suggesting high potential for inhalation and ingestion. The geoaccumulation index was generally < 1, indicating low contamination, although isolated Zn hotspots reached moderate levels. Pollution Index identified Zn as the main pollutant (max. 11.6), while Pollution Load Index classified the area as slightly polluted. Bioaccessibility tests showed negligible gastric solubility for As and Cr, while Ni, Cu, and Pb exhibited moderate to high bioaccessible fractions, with increased ingestion risk despite moderate total concentrations. SEM-EDS analysis revealed carbonaceous soot, Fe-oxide brake debris, alloy fragments, Ti–rich paint particles, and occasional As-sulfide grains in respirable sizes. Road dust showed moderate contamination but included some hotspots with bioaccessible PTEs fractions relevant to children exposure, highlighting the need for targeted dust-control measures and periodic monitoring. Simultaneously, indoor Rn concentrations in classrooms were high (mean 1540 ± 921 Bq/m^3^), posing a cancer risk by inhalation. The combined assessment of road dust and Rn exposure provided an integrated evaluation of inhalation and ingestion pathways in school environments, underscoring the importance of multi-hazard air-quality monitoring.

**Supplementary Information:**

The online version contains supplementary material available at 10.1007/s10653-026-03313-6.

## Introduction

Urban road dust is a heterogeneous mixture of crystallized minerals, metallic ions and elements, organic and inorganic compounds, and biological materials (Bartkowiak et al., 2017; Khpalwak et al., 2019). This mixture includes vehicle-derived debris (e.g., pavement, brake, tire wear) and atmospheric fallout, often containing high levels of potentially toxic elements (PTEs) such as As, Cr, Ni, and Cd. These metals enter road dust from vehicle exhaust and brake/tire abrasion, industrial deposition, and resuspended soil particles, e.g., brake-pad wear releases ultrafine particles rich in Cu, Zr, and rare earth elements (REE) (Beauchemin et al., 2021). Road-deposited dust can be resuspended by traffic and wind, and its fine particle-size fractions are therefore relevant as potential contributors to inhalation exposure, even when ambient particulate matter (PM) concentrations are not directly measured (Beauchemin et al., 2021). A high amount of dust is expected to be released by industrial and road traffic activities (Tan et al., 2018). Zheng et al. (2010) showed a correlation between PTEs concentration and regions with heavy traffic, and even after several years, metal(loids) contamination in road dust is difficult to be removed (Men et al., 2021). Industrialization leads to the production of road dust in urban areas (Khan & Strand, 2018), and taking into account the nature and source, dust can be classified as road and fugitive dust. USEPA (2010) defined road dust as solid particles that are generated by any mechanical processing of materials, including crushing, grinding, rapid impact, handling, detonation, and decrepitation of organic and inorganic materials such as rock, ore, and metal. Dust was referred to as road dust when it stays in the air, mainly due to the friction of tires moving on unpaved dirt roads and dust-covered paved roads. Fugitive dust is defined as dust that is not emitted from definable point sources, such as industrial smokestacks (Khan & Strand, 2018). Outdoor air pollution, automobile exhaust, and traffic emissions are the common exterior sources of dust (Burroughs & Hansen, 2011).

In road-deposited dust studies, particle-size fractionation is used to identify the fractions with greater potential for resuspension, inhalation, hand adhesion, and incidental ingestion, particularly the < 250 µm, < 106 µm, PM_10_-equivalent (Ø < 10 µm), and finer fractions (e.g., PM_2.5_, Ø < 2.5 µm; PM_1_, Ø < 1 µm). Traffic activity is also a major contributor to non-exhaust particles deposited on road surfaces, including brake wear, tire wear, pavement abrasion, and resuspended mineral material (Petit et al., 2019; EEA 2020; Pio et al., 2020). Over time, PTEs will accumulate in road dust, creating a contaminated reservoir that can enter the human body via breathing, ingestion, or skin contact (Li et al., 2014; Wang et al., 2025; Zheng et al., 2013). Urban areas residents are exposed to road dust PTEs mainly by inhalation of fine particles (PM_10_), by ingestion (hand-to-mouth transfer or ingestion of dust), and to a much smaller extent by dermal contact (Wang et al., 2025). For example, Cd absorption through the skin is negligible (ATSDR, 2015), and dermal absorption of Ni is also slow and limited (ATSDR, 2021a), but, direct skin contact with Ni-containing particles may cause allergic dermatitis in sensitized individuals (ATSDR, 2021a). Once entering the human body, PTEs concentrate in different organs, with inhaled ones taken by lung cells and may enter the blood system. Dust ingestion, especially through children's hand-to-mouth behaviors, transfers metals to the gut, with only a small fraction (typically < 10%) absorbed into blood, while some PTEs can bioaccumulate in tissues (ATSDR, 2018). Ingestion leads to absorption in the gastrointestinal tract, after which elements distribute differently within the body, e.g., As is widely distributed and can cross cell membranes and the placenta, while Pb can cross the blood–brain barrier and accumulate in bones and soft tissues (ATSDR, 2020). The fine and ultrafine particles have a high surface area and are especially bioavailable, they not only carry high metal loads but also penetrate deeply and can be taken by cells or via olfactory nerves into the brain (Beauchemin et al., 2021). Thus, even a small PM_10_ fraction of road dust can promote significant doses of toxic metals.

Heavy metals in road dust exert toxicity through several biochemical mechanisms. A unifying theme is oxidative stress, with many transition metals (Cu, Cr, Ni, etc.) that can participate in redox cycling, generating reactive oxygen species (ROS) that damage cellular macromolecules (Beauchemin et al., 2021). Inhaled metal-bearing particulates stimulate inflammatory responses in lung tissue, triggering cytokine release and oxidative injury. It was reported that fine particles enriched in Cd, Cr, V, and other metals readily induce ROS and inflammatory markers in bronchial cells (Beauchemin et al., 2021). Metals also bind to and inactivate critical enzymes and proteins, e.g., As (especially trivalent As(III)) has a high affinity for sulfhydryl (-SH) groups on enzymes, disrupting cellular respiration and antioxidant systems, impairing the Nrf2-mediated oxidative stress response, and leading to DNA damage (ATSDR, 2021b). Genotoxicity is another key mechanism, e.g., Cr(VI) is a well‑documented respiratory carcinogen that readily crosses cell membranes (mimicking sulfate) and then is intracellularly reduced to Cr(III), generating reactive intermediates that form DNA adducts and strand breaks; workers exposed to Cr(VI) compounds showed increased lung and nasal cancer rates (NTP, 2021). Nickel compounds also induce chromosomal aberrations and DNA mutations, with occupational studies establishing a link between chronic Ni dust inhalation and lung and nasal cancers (ATSDR, 2021a).

According to Zheng et al. (2021), particles < 250 µm adhere to hands and thus may be ingested through hand-to-mouth behavior, with dust acting as a pathway of exposure (Lu et al., 2014; Olujimi et al., 2015; Praveena et al., 2015). According to Jose and Srimuruganandam (2020), juvenile health is strongly affected by the presence of PTEs in the urban environment, particularly children, who are more vulnerable to PTEs from road dust since they spend a higher fraction of their day outdoors (Zheng et al., 2010), being exposed to dust by inhalation (Praveena et al., 2015). A large portion of urban schools are located near roads with high traffic, exposing children to vehicle emissions and traffic-generated air and dust pollution (Praveena et al., 2015). Children today spend long periods in school, so school location is an important factor in dust exposure (Meza-Figueroa et al., 2007). Children need more air than adults, causing them to breathe in more polluted air (Lu et al., 2014; Tong & Lam, 2000), and a high respiration rate due to the small body, and crawling action increases the contact and risk of children ingesting heavy metals in dust (Tong & Lam, 1998).

Regions with natural Radon gas (Rn) emissions are of major concern worldwide due to the negative impacts on indoor air quality, as the WHO (2023) classified Rn as the leading cause of lung cancer among non-smokers and the most significant natural source of ionizing radiation exposure in Europe, making indoor environments, particularly in schools, critical locations for monitoring (Cofone et al., 2025). Being colorless and odorless, Rn cannot be detected by humans. Children are especially vulnerable since their developing respiratory system is more radiosensitive and they spend extended periods indoors, often up to 8 to 10 h daily in classrooms, where ventilation may be limited, and Rn can accumulate (Kendall & Smith, 2005). Portugal implemented the 59/Euratom (2013) directive since 2018 (DR 232/2018 2018), establishing ionizing radiation guidelines with an indoor air Rn maximum of 300 Bq/m^3^. Studies reported that Rn progeny readily attach to airborne particles, including fine dust fractions (PM_2.5_, PM_10_), increasing the likelihood of inhalation and deep lung deposition (e.g., Chernyak et al., 2023; Li et al., 2018a; Yu et al., 2023). This interaction highlights the relevance of simultaneously assessing Rn and road dust dynamics near schools, as resuspended dust particles may enhance the effective dose received by children during outdoor/indoor transitions. Guarda, selected as the study area for this study, is located in a granitic region known for the naturally elevated Rn potential (Pereira et al., 2017), and has been identified in national surveys as having above-average indoor Rn levels in public buildings, including educational facilities (Antão, 2014; Lopes et al., 2018; Louro et al., 2013). Incorporating indoor Rn measurements collected simultaneously with outdoor road dust samples can provide a more comprehensive assessment of environmental hazards in and around public schools, reinforcing the need for integrated air-quality and radiation-protection strategies.

This study aims to (1) characterize outdoor road dust from areas surrounding public schools, from nurseries to secondary education; (2) quantify PTEs concentration in road dust and identify potential contamination sources; (3) assess the particle-size distribution of deposited road dust, including PM_10_-equivalent and finer fractions, as indicators of potential resuspension, inhalation relevance, hand adhesion, and ingestion exposure; (4) evaluate the potential health risk to children through PTEs bioaccessible fractions using gastric-phase extractions; and (5) incorporate indoor Rn measurements collected in the same public schools to provide a multi-hazard environmental screening of outdoor deposited dust contamination and indoor radiological exposure.

## Materials and methods

### Study area

**Fig. 1:**
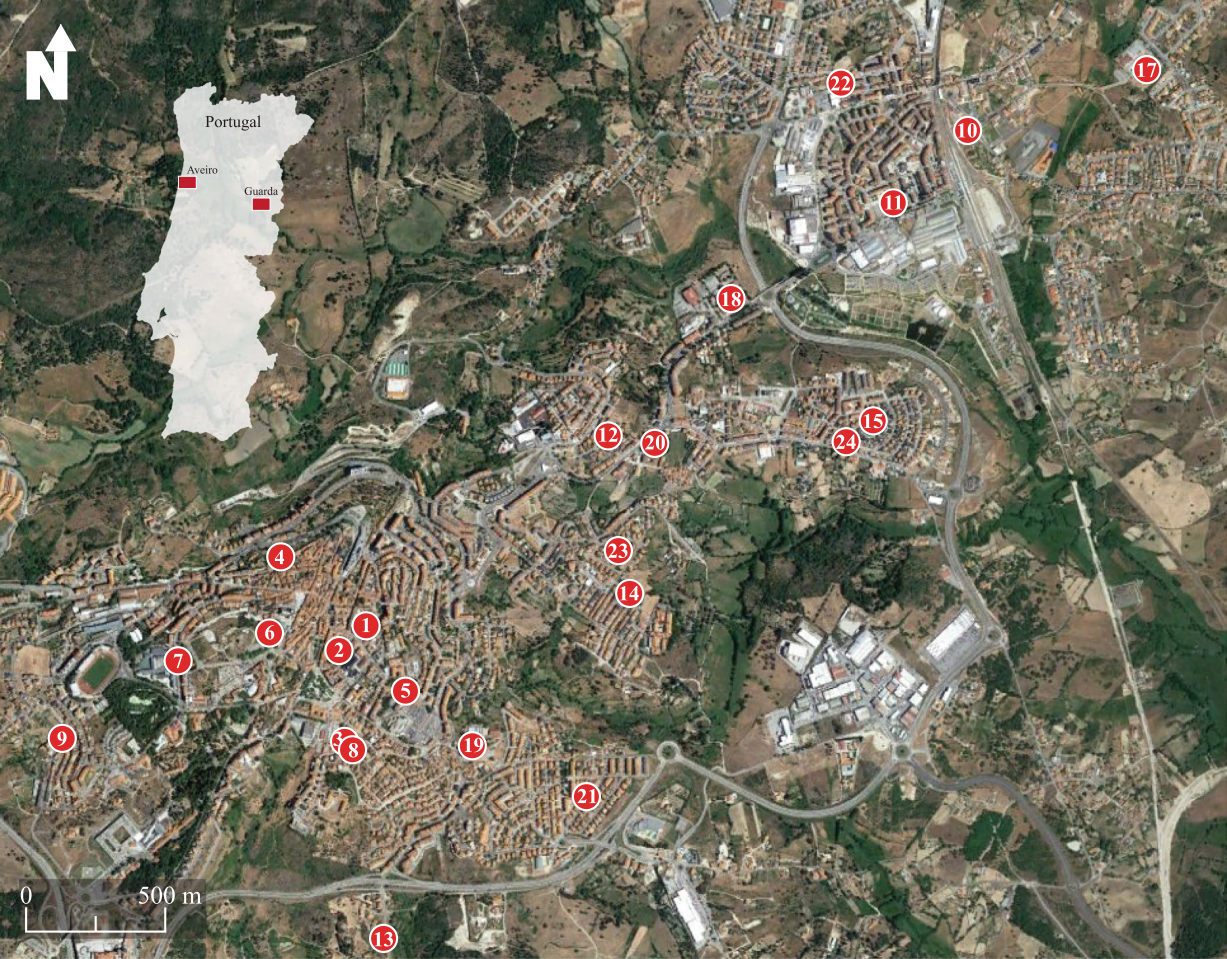
Study area and sampling location (adapt. from Google Earth Pro.®, 2025)

Guarda city is located in the center of Portugal, more specifically in Beira Alta, near the border with Spain (Fig. 1). It belongs to the sub-region of Beiras and Serra da Estrela, and constitutes the highest city in mainland Portugal, at an altitude of 1056 m (Galveias et al., 2022). It is the capital of the Guarda district, with 42,541 inhabitants. According to Galveias et al. (2022), this city has limited local industrial activity, while traffic within the city, nearby highways such as the A23 and A25, and biomass or natural-gas combustion for building heating represent possible sources of atmospheric emissions. Regional atmospheric transport from industrial areas in neighboring cities may also contribute to particle deposition.

According to Köppen Climate Classification (Miranda et al., 2001; PSAI 2022;), Guarda has a wet Mediterranean climate. According to the Portuguese Sea and Atmosphere Institute (PSAI 2022), the average annual temperature is ~ 11 °C, and the temperature variation between the warmest and coldest months is ~ 15 °C. Annual relative humidity varies from 50 to 90%, and total average annual rainfall is ~ 80 mm. Wind blows mainly from the NW quadrant (Lisboa et al., 2016; PSAI 2022).

Geologically, Guarda city belongs to the Central Iberian Zone, characterized by the predominance of granites whose intrusion occurred essentially during the Hercynian Orogeny, and Philonian rocks (Teixeira et al., 1963). Formations are essentially of granitic origin with small interspersed schist patches, numerous veins, mainly of quartz and basic rocks, and some recent alluvial deposits (Teixeira et al., 1963). The study area consists of fine-grained porphyroid granites, quartz dykes found to fill fractures, and aplite-pegmatitic veins (Teixeira et al., 1963).

### Sampling and samples characterization

In one sampling campaign, a total of seventeen road dust samples were collected in front of the entrances of public schools in Guarda city (Fig. 1), from nursery to secondary education, during the 2020 winter, labelled D1 to D17 (D: dust). Road dust samples D1, D2, D5, D16, D19, D23, and D24 were not collected due to local logistical constraints. In parallel, indoor Rn monitoring was conducted in twenty-three (except for sample D16, which was corrupted during the monitoring period; Fig. 1) schools using passive CR-39 solid-state nuclear track detectors deployed for a 3-month exposure period. Sensors were placed in the classrooms of the ground floor, 1–2 m from the floor, near the teacher so that the students wouldn’t have access, and 20 cm from the walls. Guarda is located in eastern Portugal, northeast of the Serra da Estrela, at an elevation of 1000 m above sea level, being the highest city in Portugal. During winter, ambient temperatures in Guarda are usually < 8 °C, with recurrent frost events and sub-zero nocturnal temperatures, conditions that lead classrooms and school buildings to be kept with windows predominantly closed, thereby reducing natural ventilation and favoring the accumulation of indoor Rn. In the same period, in Aveiro city (Portugal; Fig. 1), known for low natural Rn emissions (sedimentary geological setting), which was used as a control case, with monitoring of 5 public school classrooms. For each school, notes were taken describing the location and surrounding conditions (Table 1).

**Table 1:** Samples ID, traffic flow, surrounding environment, and site features

ID	Traffic flow	Surrounding environment	Site features
D1	Medium	Historic center; commerce, and restaurants	Crosswalk; roundabout in front of the school; nearby intersections
D2	Medium	Historic center; commerce, and restaurants	Crosswalk; parking areas
D3	Medium	Single-family buildings	Crosswalk; straight section after a slight curve
D4	High	Single-family buildings	Crosswalk; straight section after a slight curve
D5	Medium	Historic center; commerce, and restaurants	Crosswalk; nearby intersections
D6	High	Historic center; commerce, and restaurants	Crosswalk; roundabout
D7	High	Single-family buildings	Crosswalk; straight section after a slight curve
D8	Low	Single-family buildings	Curve
D9	Medium	Single-family buildings	Crosswalk; straight section after curve
D10	High	Single-family buildings; uninhabited areas	Crosswalk; straight section with an intersection after a curve; railway line behind the school
D11	Medium	Gas station; industrial zone	Crosswalk; straight section with traffic lights
D12	Low	Single-family buildings	Crosswalk located on a curve
D13	Low	Rural environment; few single-family buildings	Crosswalk; straight section with a speed bump
D14	Low	Single-family buildings	Crosswalk; straight section followed by a 90 º curve
D15	Medium	Single-family buildings	Crosswalk; straight section followed by a curve
D16	–––––––––––––- Not collected –––––––––––––-		
D17	Medium	Rural environment; single-family buildings	Crosswalk; straight section with intersections
D18	Medium	Multifamily buildings	Crosswalk; straight section after a roundabout
D19	Medium	Single buildings	Crosswalk; public transport stop
D20	High	Commerce and restaurants; multifamily buildings	Straight section; parking areas
D21	Low	Single-family buildings	Crosswalk; straight section
D22	Medium	Multifamily buildings	Crosswalk; straight section; parking areas
D23	Low	Multifamily buildings	Crosswalk; located on a curve
D24	Low	Single-family buildings	Crosswalk

A new, clean broom and shovel were used for each sampling site in order to avoid possible contamination. After collection, samples were stored in individually referenced polyethylene bags until preparation and analysis. In the laboratory, samples were oven dried (~ 40 °C) and subsequently quartered and divided into two representative samples, each with approximately half of the total weight. Road dust samples, < 2 mm fraction, were subjected to wet sieving for further analysis. A portion of the < 2 mm and < 250 µm fractions was grounded on an agate mill for chemical and mineralogical analysis.

*Granulometric distribution* The quantification of the granulometric size distribution of the dust fraction < 106 µm was determined using a Sedigraph III Plus X-ray grain size analyzer (Micromeritics® Instrument Corp., Norcross, GA, USA). This technique is based on the sedimentation theory (Stokes law) and the absorption of X-radiation (Beer-Lambert law) in the determination of the relative mass of a sample by particle size (Almeida et al., 2023).

*Physicochemical parameters* The pH and Electrical conductivity (EC) were measured in accordance with ISO 10390:^2021^ and ISO 11265:^1998^, respectively. Measurements were carried out using a calibrated Hanna HI 98494 multiparameter probe (Hanna Instruments Inc., Woonsocket, RI, USA; accuracy ± 0.05) in a 1:5 soil-to-distilled water suspension. The organic matter content was determined following the procedure described by Reeuwijk (2002), using the loss-on-ignition method, based on the difference in sample weight before and after burning processes.

*Mineral phases* Road dust < 2 mm mineralogy was determined by X-Ray Diffraction (XRD), using a Philips/Panalytical powder diffractometer, model X’Pert-Pro MPD, equipped with an automatic slit (Malvern Panalytical, Worcestershire, UK). A Cu-X-Ray tube was operated at 50 kV and 30 mA, which allowed data to be collected from 2 to 70° 2θ with a step size of 1° and a counting interval of 0.02 s (Sturges & Harrison, 1989).

*Chemical composition* The < 2 mm and < 250 µm fractions chemical composition was achieved by X-ray fluorescence (XRF), using a Panalytical Axios spectrometer PW4400/40 X-ray (Marvel Panalytical, Almelo, The Netherlands) operating a Rh tube under argon/methane. The limits of detection (LODs) were 0.5 mg/kg for Al, Ca, Cl, Fe, Mg, Na, S, Si, and Ti, 4.1 mg/kg for As, 2 mg/kg for Cr and Ni, 6.9 mg/kg for Ba, 0.8 mg/kg for Br and Zr, 3.9 mg/kg for Cd, 10.9 mg/kg for Ce, 2.8 mg/kg for Cu and V, 0.6 mg/kg for K and P, 1.7 mg/kg for Pb, and 1.3 mg/kg for Zn. These values correspond to the instrumental LODs automatically determined by the XRF calibration procedure and reported by the equipment software.

*Magnetic susceptibility* (χ) Magnetic properties were measured in the < 250 µm fraction using a Bartington Instruments MS2 susceptibility meter (Bartington Instruments Ltd., Oxford, UK), equipped with an MS2B dual-frequency sensor. This instrument operates at low (0.47 kHz) and high (4.7 kHz) frequencies with a sensitivity of approximately 1 × 10^–6^ SI. For each sample, magnetic susceptibility was determined at both frequencies and repeated at least three times to minimize instrumental error, following commonly applied procedures in environmental magnetism (Oudeika et al., 2020; Rachwał et al., 2017). Mass-specific susceptibility (m^3^/kg) was calculated by normalizing the measured volume susceptibility (κ) to sample mass, and frequency-dependent susceptibility (χ_fd_; %) was obtained using

$${\chi}_{fd}=\frac{{\chi}_{LF}-{\chi}_{HF}}{{\chi}_{LF}}\times 100$$

where χ_LF_ and χ_HF_ are mass-specific susceptibilities measured at low and high frequencies, respectively (Dearing, 1999; Rachwał et al., 2017). Frequency-dependent susceptibility is used to indicate the relative presence of ultrafine superparamagnetic grains (d < 0.03 µm), which respond strongly at low field frequency but weakly at high frequency, whereas increases in χ at both frequencies typically reflect the presence of coarser ferrimagnetic particles of technogenic origin (Dearing et al., 1996; Rachwał et al., 2017). The MS2 system has been widely applied in soil and dust studies as a rapid, non-destructive proxy for ferrimagnetic particle load and associated heavy-metal enrichment (Castanheiro et al., 2016; Oudeika et al., 2020).

In vitro *gastric bioaccessibility* Oral bioaccessibility of As, Cr, Cu, Pb, and Ni was performed on road dust < 250 µm fraction according to the USEPA (2007) protocol, to investigate PTEs content that can be extracted in simulated gastric fluid (SGF). Samples were extracted in a 0.4 M solution of glycine adjusted to pH 1.5 ± 0.05 by reagent-grade HCl at a L/S ratio of 100. The mixture was gently agitated for 2 h at 37 °C, and pH was regularly checked and manually adjusted by drop addition of HCl in the case of any pH drift caused by the high buffering capacity of the samples (USEPA, 2007). After extraction procedures, solutions were filtered through a membrane filter, diluted, and analyzed for metal(loid)s by Inductively Coupled Plasma Mass Spectrometry (ICP-MS). Quality control (QC) included procedural blanks, duplicate extractions, ICP-MS analytical repeats, monitoring of SGF pH before and after extraction, and verification of element-specific limit of detection (LOD) values. The corresponding QC data, including blank levels, duplicate relative standard deviation (RSD), recovery values where available, and pH stability during extraction. Subsequently, the bioaccessible fraction (%BAF) was determined by conversion of metal(loid)s concentration, in mg/kg, by

$$\%BAF=\frac{concentration \,of \,bioaccessible \,element}{concentration \,of \,total \,element \,in \,sample}\times 100$$

*Particle morphology and semi-quantitative chemical analysis* The < 250 µm fraction individual particles were analyzed with a Tescan scanning electron microscope (SEM), model VEGA LMU, which operates in high and low vacuum, coupled to an energy dispersive spectrometer (EDS) to assess morphological, granulometric, and semi-quantitative chemical properties. The identification of inorganic insoluble particles was performed using a mix of protocols for each particle (Almeida et al., 2023, 2025).

*Rn sensors*: For the analysis of the CR-39 sensors, rigorous quality-control procedures for the devices were ensured by the Laboratory of Natural Radioactivity of the University of Coimbra (https://www.uc.pt/en/fctuc/departamento-de-ciencias-da-terra/research/lrn/), accredited by the Portuguese Accreditation Institute. The sensors were etched at 90^a^C in a solution of NaOH during 4h30m, and the nuclear tracks were measured by image analysis techniques using a Radosys system. The limits of detection is of 1 Bq/m^3^ and the average combined uncertainties of 15% of the measured value.

Internal standards, certified reference material, and quality control blanks were used for monitoring the precision and accuracy of the analyses and digestion procedures. Samples revealed results were within the 95% confidence limits, and the Relative Standard Deviation was between 5 and 10%.

### Health and environmental impacts

Contamination levels were assessed in the < 2 mm fraction, widely recognized as the most relevant for pollution management studies (Gavrić et al., 2023). It should be noted that, due to the absence of specific regulatory thresholds for road dust, soil guideline values were used as screening benchmarks. Additionally, the reference value range (RVR) proposed by Sezgin et al. (2003) was adopted as a proxy for background (bkg) concentrations due to the lack of locally established background values for road dust. The RVR represents concentration intervals commonly reported for urban road dust and has been widely used as a comparative benchmark in contamination studies, providing a reference framework for interpreting contamination levels.

*Geoaccumulation Index (I*_*geo*_*)* Has been used to compare the concentration of a metal in samples and reference values (Wedepohl, 1995). I_geo_ was introduced by Müller (1969) and is calculated using the following formula:

$${I}_{geo}={log}_{2 }(\frac{{C}_{n}}{1.5*{B}_{n}})$$

where C_n_ represents the determined concentration of the toxic metal in road dust, and B_n_ denotes the reference concentration derived from the RVR values (Sezgin et al., 2003). The multiplier 1.5 was used, owing to the decrease in background value variations that may be attributed to lithogenic variations. According to Müller (1969) and Ali et al. (2017), the I_geo_ values are divided into seven groups: I_geo_ ≤ 0 = uncontaminated; 0 < I_geo_ ≤ 1 = uncontaminated to moderately contaminated; 1 < I_geo_ ≤ 2 = moderately contaminated; 2 < I_geo_ ≤ 3 = moderately to heavily contaminated; 3 < I_geo_ ≤ 4 = heavily contaminated; 4 < I_geo_ ≤ 5 = heavily to extremely contaminated; and I_geo_ > 5 = extremely contaminated.

*Pollution Index (PI)* Is useful for basic estimation of a sample’s relative degree of pollution of a single metal and is calculated as follows:

$${PI}_{i}={\left(Ci\right)}_{s}/{(Ci)}_{bkg}$$

where, (Ci)_s_ is the concentration of metal *i* in the sample, and (Ci)_bkg_ corresponds to the reference value obtained from Sezgin et al. (2003). According to Chen et al. (2005), the pollution level is categorized as follows: PI ≤ 1 is Low, 1 < PI ≤ 3 is Mid-Level, and PI > 3 is High.

*Pollution Load Index (PLI)*: Is used as a metric to assess the overall pollutant loading in sediment, soil, and road dust (Chen et al., 2015; Gloaguen et al., 2021). Since this indicator considers the level of metal contamination from several metals, it is a better index of the overall pollution status of a sampling site. PLI is calculated as follows:

$$PLI={({PI}_{1}*{PI}_{2}* {PI}_{3}*\dots * {PI}_{n})}^{1/n}$$

where, PI_1_, PI_2_, and PI_3_ are the PI of heavy metals 1, 2, and 3, respectively, and *n* is the number of trace metals (Tomlinson et al., 1980). Based on PLI, road dust samples can be categorized as follows: unpolluted: PLI ≤ 1; slightly polluted: 1 < PLI ≤ 2; moderated polluted: 2 < PLI ≤ 3; and heavily polluted: PLI ≥ 3 (Chen et al., 2015; Liu et al., 2016).

*Rn inhalation cancer risk*: To calculate the inhalation cancer risk associated with exposure to Rn among students attending the educational facilities, the method defined by the USEPA (2023, 2025) was applied, using the equation *Risk* = *CDI* × *SF*, where Risk represents the probability of an individual developing cancer over a lifetime (dimensionless); CDI = (C × IR × EF × ED) / (AT), is the chronic daily intake (pCi·year/m^3^); and SF the slope factor (risk per m^3^/pCi·year). The indoor exposure factors relevant to students were considered: exposure duration (ED, years) = 12; exposure frequency (EF, days/year) = 100; conservative exposure time (ET, hours/day) = 10; gaseous saturation factor (GSF, dimensionless) = 1; inhalation rate (IRA, m^3^) = 60; exposure time (t, years) = 10; inhalation risk factor (risk/Bq) = 6.16E-11; and external exposure factor (risk/year per Bq/m^3^) = 4.39E-11. Calculations were performed using the USEPA RAIS (Risk Assessment Information System) calculator (USEPA, 2025). Cancer risk is a probability of effects, expressed as a fraction, without units, from 0 to 1.0, where a probability of 1.0 implies an absolute certainty that an event or outcome will occur, and, e.g., 1.00E-04 denotes that one person in ten thousand will develop cancer. The risk thresholds are: (a) Risk < 1.00E-6, indicating no significant risk; (b) 1.00E-6 < Risk < 1.00E-4, indicating that mitigation measures should be implemented; and (c) Risk > 1.00E-4, indicating a high and unacceptable risk (USEPA, 2025).

## Results and discussion

### Outdoor road dust

#### Physicochemical properties

The physicochemical properties of road dust, particularly pH, electrical conductivity (EC), and organic matter (OM), are key parameters influencing the reactivity, retention, and mobility of potentially toxic elements (PTEs) within urban environments. These properties largely reflect the combination of local lithology, traffic-related emissions, road maintenance practices, and weathering processes acting on the dust matrix (Kasimov et al., 2019). The studied samples exhibited pH values ranging from 5.85 to 7.02 (< 2 mm) and 6.60 to 8.41 (< 250 µm), with average values of 6.43 ± 0.37 and 7.40 ± 0.53, respectively (Table 2). The < 106 µm fraction showed intermediate but overall near-neutral pH values (6.71–7.61; mean 7.20 ± 0.30). According to Luo et al. (2008), pH is one of the most influential parameters controlling PTEs mobility in dust and soils, as it regulates metal solubility, sorption capacity, and surface charge of particulate matter. The progressive shift toward neutral to slightly alkaline conditions with decreasing particle size was consistent with patterns described in urban environments dominated by vehicular abrasion and road maintenance inputs (Kasimov et al., 2019). Fine road dust tends to accumulate carbonate-rich particles, asphalt wear debris, cementitious fragments, and salt-derived residues, which collectively elevate pH. Comparable trends were observed in Chinese campus road dust with pH ranging 5.7–11.2 (Chen et al., 2014) and urban-industrial environments in Greece with pH 7.4–8.9 (Botsou et al., 2022). In Guarda, characterized by cold winters, frequent use of de-icing salts, and mechanical abrasion from tires under low-temperature conditions, these patterns were expected. Kasimov et al. (2019) demonstrated that salting practices and abrasion products significantly increase dust alkalinity during winter, especially along main circulation routes. This behavior mirrors what was observed in the present study, with the < 250 µm fraction, the one most susceptible to de-icing salt accumulation and resuspension, exhibiting the highest pH values. From a geochemical perspective, this increase in alkalinity can favor the sorption and retention of divalent metal cations (Pb^2+^, Zn^2+^, Cu^2+^), decreasing their immediate mobility (Vlasov et al., 2022). In contrast, oxyanion-forming elements (e.g., As, Mo, Sb) may become more mobile under alkaline conditions, depending on their specific speciation and competitive adsorption processes (Adriano, 2001). Overall, the pH behavior of the samples suits well within the global urban dust ranges and reflects typical influences of winter maintenance and traffic-related abrasion.

**Table 2:** Physicochemical properties of the < 2 mm, < 250 µm, and < 106 µm fractions

	< 2 mm			< 250 µm		< 106 µm	
ID	pH	EC	OM	pH	EC	pH	EC
Minimum	5.85	2378	0.6	6.60	33.1	6.71	16.2
Maximum	7.02	2985	11.4	8.41	416	7.61	201
Mean ± SD	6.43 ± 0.37	2737 ± 163.5	3.8 ± 0.03	7.40 ± 0.53	174 ± 119	7.20 ± 0.30	80.8 ± 67.2

EC – electrical conductivity (µS/cm); OM – organic matter (%); SD – standard deviation

The < 2 mm fraction showed high EC (2378–2985 µS/cm), whereas the < 250 µm fraction presented lower values (33.1–416 µS/cm), and the finest < 106 µm fraction displayed intermediate EC (16.2–201 µS/cm) (Table 2). The strong contrast between coarser and finer fractions showed that coarser road dust retained substantial amounts of soluble ions, including chlorides, sulphates, carbonates, and alkali metals, consistent with the incorporation of, e.g., road maintenance materials (salt, sanding aggregates), concrete and cement-derived particles (Ca^2+^, Mg^2+^, carbonate), and soil-derived crustal inputs. This interpretation agrees with observations made for high-altitude Latin American cities by Vanegas et al. (2021), who reported that coarser road dust often preserves higher loads of salts and weathered pavement debris when compared to PM_10_. The lower EC in the finer fractions was consistent with the higher mineral purity and lower content of readily soluble salts. Finer dust is usually, dominated by silicates, aluminosilicates, soot, tire wear, and resuspended PM_10_, which typically exhibit modest conductivity unless enriched by anthropogenic salts. Kasimov et al. (2019) highlighted similar behavior in Moscow dust, where EC increased progressively within heavy-traffic arterial roads due to repeated application of de-icing agents and accumulation of road abrasion residues. Although the schools sampled in Guarda were located mainly in residential areas, EC values, particularly in the < 2 mm fraction, suggested the influence of winter maintenance and crustal contributions.

Organic matter content plays an important role in regulating the binding, complexation, and potential immobilization of PTEs, providing reactive functional groups (carboxyl, phenolic, hydroxyl) capable of sorbing oxyanions and cations (Gandois et al., 2010; Tang et al., 2014). In the < 2 mm fraction, OM ranged widely (0.6–11.4%; mean 3.8%), revealing significant variation between sampling locations. Samples with elevated OM (notably D9) corresponded to an area near traffic-slowing features, curved roads, and pedestrian-school interactions, which tend to accumulate exhaust-derived soot and unburned hydrocarbons, tire and brake wear particles, microplastics, and asphalt-derived organic residues (Faure et al., 2000; Wu & Lu, 2018). These anthropogenic carbonaceous materials are known to preferentially accumulate in micro-depressions and low-speed traffic zones. The pattern observed in D9 corresponded to the results reported by Wu and Lu (2018), which detected OM up to 18.8% in dust collected near schools, parks, and bus stops due to repeated braking and low-speed manoeuvres. Finer fractions (< 250 and < 106 µm) were expected to contain a higher proportion of combustion-derived OM, which often presents low solubility and high aromaticity, enhancing their capacity to bind metals via π-electron interactions (Adriano, 2001). Li et al. (2016) demonstrated that OM strongly influences the sorption–desorption cycling of PTEs, with fine OM-rich particles acting as reservoirs for Pb, Cu, and Zn in urban dust. The elevated OM values observed in some Guarda samples explicitly reflected greater contributions from anthropogenic carbonaceous particles, which dominate fine PM in school-adjacent and residential microenvironments. Overall, the combined behavior of pH, EC, and OM in the road dust from Guarda was consistent with patterns observed in urban environments influenced by traffic, seasonal road maintenance, and mixed natural-anthropogenic particulate sources.

#### Identified mineral phases

**Fig. 2:**
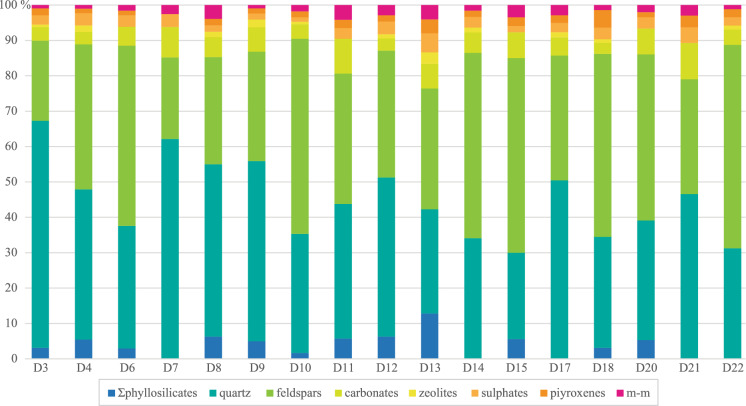
Relative distribution of the identified mineral phases in road dust samples; m-m—magnetite-maghemite

The mineralogical composition of the < 2 mm road dust fraction reflected a combination of local geological inputs, pavement materials, and traffic-related abrasion products, all of which operated under chemical conditions influenced by pH, EC, and OM. These factors collectively determined the degree of weathering, solubility, and reactivity of road dust particles (Amato et al., 2011; Gunawardana et al., 2012). Identified mineral phases on the road dust < 2 mm fraction revealed the presence of Σphyllosilicates (e.g., micas, kaolinite), quartz (SiO_2_), feldspars (K-feldspars (KAlSi_3_O_8_), plagioclases (Na,Ca)[(Si,Al)AlSi]_2_(O_8_)), carbonates (calcite (CaCO_3_), dolomite (CaMg(CO_3_)_2_), pyroaurite (Mg_6_Fe^3+^_2_(CO^3^)(OH)_16_·4(H_2_O)) and siderite (FeCO_3_)), zeolites, sulphates (anhydrite (CaSO_4_), pyroxenes (XY(Si,Al)2O6)), and magnetite-maghemite (Fe_3_O_4_-y-Fe_2_O_3_), with proportions varying between samples (Fig. 2). Σphyllosilicates represent the total content of sheet-silicate minerals, including clays (e.g., illite, smectite, kaolinite, chlorite), micas (e.g., muscovite, biotite, phlogopite), and other lamellar silicates that share similar structural, weathering, and geochemical properties, being characteristic of soil inputs, weathered granitic substrates, and degradation of building materials, as commonly observed in urban road dust (Gunawardana et al., 2012). These minerals were absent in D7, D14, D17, D21, and D22, suggesting that these sites were dominated by coarser, mechanically derived particles with limited clay enrichment. Σphyllosilicates possess high surface reactivity, which increases dust buffering capacity and enhances the sorption of cations and organic compounds (Adriano, 2001), complementing the slightly acidic to neutral pH observed for the < 2 mm fraction (mean pH 6.43 ± 0.37).

Quartz and feldspars (K-feldspars and plagioclases) constituted the dominant mineral phases across all samples, with quartz ranging from 24 to 64% and feldspars from 22 to 57%. These leucocratic minerals were consistent with the granitic and metasedimentary geology of the Guarda region, where quartz-feldspar assemblages are abundant in both natural soils and urban stonework (Pereira et al., 2018). Their high mechanical resilience can explain their prevalence in road dust exposed to tire abrasion, winter sanding, and steep-slope vehicular maneuvering (Amato et al., 2009; Candeias et al., 2020). The chemical inertness of quartz and feldspars aligned with the moderate pH and relatively low soluble EC contributions in the < 250 and < 106 µm fractions, where their high abundance limits the release of ionic species. Carbonates were detected in most samples at low to moderate levels (3–11%), being recognized as products of cement degradation, concrete dust, and road marking materials, as well as local soil inputs, particularly in urban environments undergoing continuous surface wear (Amato et al., 2009). Their presence was consistent with the observed alkaline shift in the finer fractions (pH up to 8.41), as partial dissolution of calcite and dolomite elevates pH. The high EC of the < 2 mm fraction further reflected the presence of carbonate and sulphate dissolution products (Ca^2+^, Mg^2+^, SO_4_^2−^), retained preferentially in coarser, less-weathered debris (Vanegas et al., 2021). The occurrence of siderite and pyroaurite suggested a mixed pedogenic and anthropogenic origin, including soil micro-reduction zones and corrosion products from vehicle components and metallic urban infrastructure (Liu et al., 2019). Their coexistence with elevated EC in the coarse fraction supported the interpretation that ion-rich crustal particles and corroded materials accumulate within the < 2 mm size range. Minor zeolites and sulphates indicated additional contributions from construction materials, gypsum-containing debris, and atmospheric SO_2_/SO_4_ deposition (Somasundaram et al., 2015). These minerals can contribute to ionic enrichment and surface alkalinity, but are present in quantities too low to dominate chemical behavior. Pyroxenes, identified in most samples, are typical constituents of pavement aggregates, quarry-derived gravel, and local granitoids, confirming mechanical fragmentation as a major dust source. Their presence aligned with high quartz and feldspar contents, reflecting the mineralogical signature of regional granitoids and urban stone pavements. Ferrimagnetic magnetite-maghemite (up to 4.1%) represented the environmentally significant anthropogenic component. These Fe-oxides are well-established markers of non-exhaust vehicular emissions, particularly brake wear, exhaust nano-oxides, and abrasion of metallic vehicle parts (Hofman et al., 2017; Li et al., 2016). Their co-occurrence with elevated OM in the finer fractions and with moderate EC suggested associations with carbonaceous traffic-derived particles, enabling sorption of metals such as Cu, Zn, and Pb. The mineral assemblage of the < 2 mm fraction supported the physicochemical patterns of the studied dust. The coarse fraction, enriched in carbonates, sulphates, and corroded metallic phases, showing high EC and moderate pH, whereas the finer fractions, rich in Σphyllosilicates, quartz, feldspars, and magnetite-maghemite, exhibiting higher OM, lower EC, and near-neutral to alkaline pH. This combined behavior indicated that road dust composition in Guarda was derived from the interaction of local granite-derived materials, construction-related inputs, and traffic non-exhaust emissions, modulated by winter maintenance practices and urban microenvironmental dynamics.

#### Major elements composition

The major elements composition of the < 2 mm road dust fraction was dominated by silicate-bound elements, with Si and Al as the most abundant components, followed by K, Fe, Ca, and Na, and minor Mg, Ti, P, S, and Cl (Table 3). Mean concentrations were 31.61 ± 2.63% for Si, 6.71 ± 1.01% for Al, 5.06 ± 0.67% for K, 1.43 ± 0.34% for Fe, 1.30 ± 0.28% for Na, 1.20 ± 0.60% for Ca, and 0.32 ± 0.07% for Mg, with Ti averaging 0.20 ± 0.04% and loss of ignition (LOI) 6.36 ± 4.93%. This composition is typical of road dust dominated by quartz-feldspars-Σphyllosilicates assemblages, consistent with the identified mineral phases on the < 2 mm fraction, and comparable to crustal signatures reported for urban road-deposited sediments in other urban areas (e.g., Candeias et al., 2020; Gunawardana et al., 2012; Haynes et al., 2020). The high Si and Al contents reflected the prevalence of silicate minerals (quartz, feldspars, and Σphyllosilicates) derived from local soils, weathered bedrock, and construction stones. The Guarda region is underlain by granitic and metasedimentary rocks, which are naturally rich in quartz and K-feldspars, with secondary plagioclase and micas. This lithological context was reflected by the high Si and Al content in the road dust samples. Comparable dominance of silicate-bound Si and Al was reported in road dust in e.g., Australia, and China, where SiO_2_ and Al_2_O_3_ accounted for most of the bulk composition (often > 70%) (Gunawardana et al., 2012; Yang et al., 2015). Potassium and Na were present at levels consistent with alkali feldspars and micas as major phases, in agreement with the XRD identification of K-feldspar, plagioclase, and Σphyllosilicates in the < 2 mm fraction. Similar K and Na contents were reported in road-deposited sediments, where K and Na were largely controlled by feldspathic minerals and showed limited anthropogenic enrichment relative to local soils (Gunawardana et al., 2012; Yang et al., 2015). Iron and Mg content mainly reflected contributions from ferromagnesian silicates and Fe-oxide phases, consistent with the presence of magnetite-maghemite and pyroxenes (Fig. 2). The Fe content was within the range reported for road dust in other studies, where Fe_2_O_3_ typically varied between 3 and 6% as oxides (equivalent to ~ 2–4% Fe) (Li et al., 2018b; Moskovchenko et al., 2022). In urban environments, Fe is partly crustal but also associated with traffic-related Fe-bearing particles (brake wear, steel corrosion, rail and pavement abrasion), known to contribute magnetite-maghemite to the dust load (Candeias et al., 2020; Hofman et al., 2017). The relatively modest dispersion in Fe concentrations across samples suggested a combined, but spatially consistent, influence of local geology and vehicular non-exhaust emissions. Calcium displayed a wider range (Table 3) and the highest relative variability among major elements, typical of urban dust influenced by cementitious materials, concrete, mortar, plaster, and road-marking paints. Studies reported that CaO in urban and road dust exhibited larger spatial variability than other oxides, largely due to variable inputs from construction and demolition debris, concrete surfaces, and calcareous fillers (Švédová et al., 2020; Yang et al., 2015). In the present study, the Ca range was consistent with low to moderate carbonate content (Fig. 2), suggesting that both construction-related carbonates and natural carbonates contributed to Ca enrichment in the < 2 mm fraction. Chlorine, P, and S occurred at low levels (Table 3). These elements are generally associated with soluble salts, fertilizer residues, vehicular emissions, and gypsum/cement additives in road environments (Haynes et al., 2020; Moskovchenko et al., 2022). Chlorine has often been linked to de-icing salts and road maintenance in cold climates, sulfur to sulphate aerosols and gypsum-bearing construction materials, and phosphorous to fertilizers, dust, soil OM, and biological inputs. Although present in minor proportions, these elements may influence EC and surface reactivity, especially in the coarser fraction, where soluble phases are less washed away. Titanium content was low (0.11–0.29%), in line with typical crustal levels, and most likely hosted in Ti-bearing heavy minerals. Similar Ti abundances, often used as a conservative lithogenic marker in road dust and soils, have been reported in studies from Changchun (China), Thessaloniki (Greece) and different European cities, where Ti showed minimal anthropogenic enrichment and could be used as a reference for normalization of trace metals (Bourliva et al., 2022; Haynes et al., 2020; Yang et al., 2015). Loss on ignition (LOI) showed the widest range (1.68–19.70%), reflecting variable amounts of OM, carbonates, and structurally bound water in clays and hydroxides. High LOI values typically agree with increased contributions from asphalt-derived organics, soot, tire wear, and fine carbonaceous particles, as well as carbonates from cement and concrete dust (Candeias et al., 2020; Faure et al., 2000; Haynes et al., 2020). This variability suggested heterogeneous mixing of crustal particles with anthropogenic materials across the sampled school environments.

**Table 3:** Major elements concentration in the road dust samples statistical summary (n = 17) of the < 2 mm fraction (in %)

ID	Al	Ca	Cl	Fe	K	Mg	Na	P	S	Si	Ti	LOI
Minimum	5.56	0.41	0.01	0.87	4.09	0.21	0.89	0.1	0.01	25.54	0.11	1.68
Maximum	9.80	2.34	0.11	2.18	6.58	0.47	1.96	0.3	0.33	35.81	0.29	19.70
Mean ± SD	6.71 ± 1.01	1.20 ± 0.60	0.03 ± 0.02	1.43 ± 0.34	5.06 ± 0.67	0.32 ± 0.07	1.30 ± 0.28	0.16 ± 0.06	0.09 ± 0.08	31.61 ± 26.3	0.20 ± 0.04	6.36 ± 4.93

LOI – Loss of ignition; SD – Standard deviation

#### Total concentration of potential toxic elements

The distribution of trace and PTEs, in the < 2 mm road dust fraction, reflected both geogenic contribution and anthropogenic inputs, especially those associated with traffic activity (Table 4), with mean concentrations ranked Ba > Zn > Zr > Pb > Ce > Cu > Cr > V > As > Br > Ni > Cd. Comparable findings have been reported, identifying Zn, Cu, Pb, and Ba as the most elevated traffic-associated elements in urban environments (Chen et al., 2024; Moskovchenko et al., 2022). The Canadian soil guideline (CCME, 2025) for inorganic As in residential/parkland soils is 12 mg/kg, being a mean As content in road dust of 8.8 mg/kg, falling below the threshold, although the maximum value (29.3 mg/kg) exceeded it, suggesting local anthropogenic enrichment (Jadoon et al., 2020). Cadmium and Pb content were below CCME (2025) limits of ~ 10 and 140 mg/kg, respectively. Zn showed a range between 39.9 and 580 mg/kg, and a mean concentration also below the soil guideline of 250 mg/kg, although the maximum value exceeded it. These results suggested low to moderate contamination across most locations, with possible Zn and As hotspots requiring further analysis. Elemental groups revealed their likely origin, with Cr, Ni, V, Ce, and Zr displaying concentration ranges characteristic of lithogenic inputs, reflecting the mineralogy of local granitic-metasedimentary substrates and soil resuspension. Studies in urban environments confirmed that these elements typically follow geologic patterns unless affected by strong industrial activities (Dehghani et al., 2018). Barium showed mixed origin, while naturally present in K-feldspar and micas, it is also a major tracer of brake wear, since modern brake pads frequently incorporate barite (BaSO_4_) (Sinha et al., 2020). The Cu, Zn, Pb, Cd, and Br showed typical traffic-related signatures, with Cu usually linked to brake wear, Zn to tire abrasion, lubricants, and galvanized components, Pb to legacy contamination, Cd to oils and tire wear, and Br to halogenated compounds (Amato et al., 2014; Grigoratos & Martini, 2015; Kefeni et al., 2011; Lewis et al., 2019; Thorpe & Harrison, 2008). The Zn and Cu were recognized as key non-exhaust traffic emission markers, and their strong variability across samples indicated differences in braking intensity, street geometry, and vehicular flow around the sampled schools (Fussel et al., 2022). The physicochemical conditions of the < 2 mm fraction, near-neutral pH, high EC, and moderate OM (Table 2), influenced the behavior of these PTEs, since at neutral pH, cationic metals (Pb, Cu, Zn, Cd) tend to form stable complexes with clay surfaces and organic ligands, reducing their solubility. The high EC reflects the presence of soluble chlorides, sulphates and carbonates that can either enhance metal mobility or promote precipitation (e.g., as carbonates or hydroxides). Studies of road dust and urban soils reported that salinity can increase the redistribution of Zn, Cu, and Pb during wetting–drying cycles, especially in environments with winter salting or cement-derived particulates (Acosta et al., 2011; Norrström & Bergstedt, 2001). The As species showed strong affinity for Fe hydroxides and phyllosilicates at near-neutral pH, which may limit mobility but can be reversed under competitive anions (e.g., phosphate) (Goh & Lim, 2004; Goldberg & Johnston, 2001). From a health and environmental perspective, even though total PTE concentrations in the < 2 mm fraction were mostly within guideline ranges, recent studies showed that bulk concentrations poorly predict human exposure, which can be driven by the bioaccessible portion of metals in smaller fractions (PM_10_, PM_2.5_, < 100 µm). Chen et al. (2024) reported that Zn, Cu, Pb, and As in road dust posed moderate chronic ingestion risk for children even where total concentrations were below guideline thresholds, due to high gastric bioaccessibility. Given the clear presence of traffic-related metals and the chemical conditions supporting retention of fine particulates, the subsequent assessment of < 106 µm texture, and < 250 µm magnetic susceptibility, and bioaccessible fractions of these PTEs will be essential to quantify the real exposure risk for children in Guarda.

**Table 4:** Potentially toxic elements concentration in the road dust samples statistical summary (n = 17) of the < 2 mm fraction (in mg/kg)

ID	As	Ba	Br	Cd	Ce	Cr	Cu	Ni	Pb	V	Zn	Zr
Minimum	2.1	160.0	0.9	2.0	13.5	8.6	11.0	1.0	20.1	6.3	39.9	57.3
Maximum	29.3	350.0	14.5	4.1	43.2	33.7	33.7	6.0	39.5	20.9	580.0	180.0
Mean ± SD	8.8 ± 7.1	251.2 ± 49.5	6.2 ± 3.8	2.2 ± 0.7	23.2 ± 8.1	15.7 ± 7.3	17.7 ± 5.6	3.7 ± 1.3	29.2 ± 5.7	12.4 ± 3.9	160.0 ± 156.1	88.1 ± 41.5
LOD	1.8	6.9	0.8	1.6	10.9	2.0	2.8	0.8	1.7	2.8	1.3	0.8

SD – Standard deviation; LOD – Limit of detection

#### Contamination indices of the PTEs total chemical composition

To evaluate road dust (< 2 mm fraction) geoaccumulation index (I_geo_), pollution index (PI), and pollution load index (PLI) were estimated (Table 5), considering the reference values range (RVR) proposed by Sezgin et al. (2003). The I_geo_ results indicated that for most elements the index lies below 1 (mean 0.44), which falls into the uncontaminated to moderately contaminated class. Only in some samples I_geo_, due to Zn, was > 1, being classified as moderately contaminated (D9, D17, and D20, ranging 1.02–1.85), and moderately to heavily contaminated (D15, 2.33) categories. The PI results also highlighted Zn as the dominant pollutant, with samples D4, D9, D11, D15, D17, and D29 displaying PI > 3 (ranging from 3.60 to 11.60), being classified as highly polluted. The PI for Pb revealed that only one sample (D18) was classified with a low pollution level (PI = 1.0), and the remaining with a mid-level of pollution, ranging from 1.2 to 2.0. All the other samples were classified with low pollution, except PI for Cu in samples D8, D9, D10, D11, D20, and D21 (1.05 to 1.45), and PI for As in samples D17 and D22 (1.01 and 1.47), classified with mid-level pollution. The overall PLI mean of 1.42 classified the road dust samples set in the slightly polluted range (1 < PLI ≤ 2). These findings point to a generally moderate heavy‐metal load in the road dust of the present study area, but with discrete hot spots of concern. Comparatively, other urban road dust studies showed higher contamination loads, for example, in urban Havana (Cuba), where the mean Zn concentration reached 548 mg/kg, with multiple sampling stations presenting high PI/PLI indices, indicating significant contamination near construction and traffic zones (Rizo et al., 2023). In Cairo (Egypt), an I_geo_ mean for Zn of 1.34 and peak PLI values in major traffic corridors confirmed the role of vehicle emissions and road‐dust resuspension in heavy‐metal accumulation (Mostafa et al., 2024). In Jinan (China), an inland city, the I_geo_ ranged − 6.07 to 2.67, PI reached 9.58, and the mean PLI was 1.03 (Asgari et al., 2025). Compared to these studies, the moderate values of Guarda road dust samples reflected a less‐intense urban-industrial/traffic context, yet the presence of Zn hotspots suggested a need for localized mitigation. Consistent with these international studies, the elevated Zn (and to a lesser extent Cu) is likely attributable to tire/brake wear, engine emission, and road surface abrasion. Heidari et al. (2021) found high Zn and Cu in road dust of Bandar Abbas (Iran) linked to traffic emissions. Although soil guideline values provide a useful reference framework, caution is required when applying them to road dust, as differences in particle size distribution and metal bioaccessibility may influence actual exposure (Liang et al., 2025). Therefore, exceedances of these guideline values should be interpreted as indicative of potential concern rather than definitive evidence of health risk. The use of soil-based benchmarks in this study is intended to contextualize contamination levels rather than to provide a quantitative risk assessment.

**Table 5:** Geoaccumulation index (_Igeo_), pollution index (PI), and pollution load index (PLI) of the road dust samples

	As	Cr	Cu	Pb	Zn	PLI
I_geo_	0.02-0.29	0.04-0.14	0.11-0.29	0.20-0.40	0.16-2.33	
PI	0.11-1.47	0.18-0.68	0.55-1.45	1.00-2.00	0.80-11.60	1.26-1.72

#### Magnetic susceptibility as an indicator of contamination sources

**Fig. 3:**
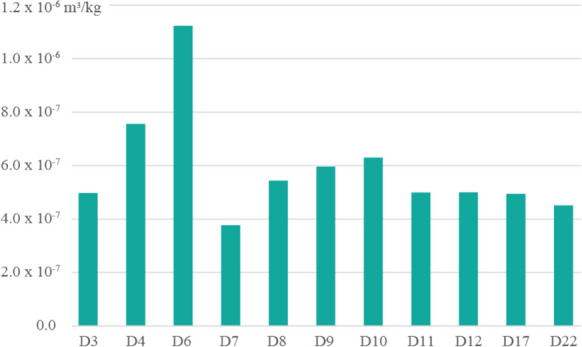
Mass-specific magnetic susceptibility (χ) of the < 250 µm road dust fraction in the selected samples (in m^3^/kg)

Mass-specific magnetic susceptibility (χ) of the < 250 µm fraction ranged from 3.78 × 10^–7^ to 1.12 × 10^–6^ m^3^/kg, with a mean of ≈5.97 × 10^–7^ m^3^/kg (excluding samples D13, D14, D15, D18 and D21, which were not analyzed due to insufficient material) (Fig. 3). Magnetic properties have been commonly used as proxies for pollution (Aguilera et al. 2026). In this study, the χ values exceeded typical background levels for uncontaminated soils (commonly ~ 10^–8^-10^–7^ m^3^/kg) and fall within the range reported for road dust and urban topsoils influenced by traffic- and combustion-derived technogenic magnetic particles (TMPs) (Vodyanitskii & Shoba, 2015; Wang et al., 2012).This suggested that, in a medium-sized city like Guarda, the fine road dust fraction carries a measurable anthropogenic ferrimagnetic signal, as noted by Aguilera et al. (2026), who highlighted that the hazards associated with magnetic particles are primarily linked to the smallest size fractions. Environmental magnetism studies showed that low-field magnetic susceptibility in urban environments is dominated by ferrimagnetic Fe-oxides (e.g., magnetite, maghemite) associated with traffic emissions, brake wear, tire abrasion, fly ash, and industrial particulates, whereas diamagnetic (quartz, carbonates) and paramagnetic (phyllosilicates, Fe-bearing silicates) minerals contribute much less to χ (Vasiliev et al., 2020; Vodyanitskii & Shoba, 2015). The identified mineral assemblage, which included magnetite-maghemite as a minor but ubiquitous phase, together with abundant quartz and feldspars, and subsidiary Σphyllosilicates and carbonates, was consistent with the observed χ values, with the bulk minerals highly diluting the magnetic signal. In contrast, a comparatively small amount of ferrimagnetic minerals controlled the susceptibility response (Vodyanitskii & Shoba, 2015; Wang et al., 2012). The magnitude of χ was comparable to values reported for road-deposited sediments in urban areas without intense industry, where mass susceptibility for fine dust fractions was in the 10^–7^-10^–6^ m^3^/kg range. Jordanova et al. (2014) found similar χ levels in road dust and showed that they increase with population size and NO_2_ concentrations, reflecting traffic intensity and urban emissions. Wang et al. (2012) reported higher χ values in street dust in heavily industrial districts, where ferrimagnetic concentrations and heavy metal contents (Fe, As, Cu, Mn, Ni, Pb, Zn) were tightly correlated. Compared to those industrial hotspots, the Guarda dust χ values suggested moderate loading of TMPs, consistent with a traffic-dominated rather than heavy-industrial emission setting around schools. Within the study area, χ variability (≈3.8–11.2 × 10^–7^ m^3^/kg) likely reflected differences in local traffic conditions, micro-topography, and dust retention. Samples D6 and D4 showed the highest mass susceptibility (1.12 × 10^–6^ and 7.56 × 10^–7^ m^3^/kg, respectively), whereas D7 and D22 displayed the lowest values (3.78 × 10^–7^ and 4.51 × 10^–7^ m^3^/kg, respectively). Higher χ in D4 and D6 corresponded to relatively elevated EC in the < 106 µm fraction and substantial PM_2_ and PM_0.1_ proportions, indicating intense deposition of fine, salt- and metal-bearing particulates. The lower χ in D7 and D22 was consistent with lower EC and a mineralogy dominated by quartz-feldspars with less magnetite-maghemite and fewer Σphyllosilicates, indicating weaker input of traffic-derived ferrimagnetic particles at those locations. This pattern reflected observations from other urban areas where χ hotspots aligned with busy junctions, bus stops, and braking/acceleration zones, while quieter residential stretches showed lower magnetic enhancement (Jordanova et al., 2014). The moderate OM content and near-neutral pH of the < 2 mm fraction suggest that TMPs in the Guarda dust are embedded in a matrix capable of retaining metal(loid)s on Fe-oxide and organic surfaces. Studies reported that χ in urban soils and dust correlated positively with Fe, Mn, and traffic-related metals, such as Cu, Zn, and Pb, because these metals are often concentrated in or on ferrimagnetic particles derived from brake pads, tires, and combustion residues (Norouzi et al., 2016; Vodyanitskii & Shoba, 2015; Wang et al., 2024). Wang et al. (2024) and Alpofead and Davidson (2025) showed that samples with higher χ tend to exhibit higher total and bioaccessible metal content, particularly for Zn, Pb, and Cu, and that magnetic parameters can be used to assess dust samples with high toxic metal(s) bioaccessibility. The combination of measurable χ enhancement, the presence of magnetite-maghemite, and elevated Zn, Cu, and Pb in the < 2 mm fraction indicated that χ is a robust proxy for anthropogenic metal-bearing particulates in these school-adjacent road dusts. The χ data confirmed that all studied samples were impacted by traffic-related technogenic magnetic particles, but with varying intensity. Samples with higher χ likely corresponded to areas where braking, idling, and turbulence formation around buildings promoted the accumulation of fine ferrimagnetic dust and associated PTEs, similarly to reports that showed that the use of simple low-field susceptibility measurements can be used as a rapid, non-destructive screening tool for spatial mapping of metal pollution in urban road dust and topsoils (Ma et al., 2022; Wang et al., 2024).

Considering the pollution indices and the magnetic susceptibility, the results indicate the same general pattern of anthropogenic influence, but they do not describe the same component of the dust. The PI, I_geo_, and PLI quantify chemical enrichment and identify the elements driving contamination, showing low to moderate overall contamination, with Zn as the main pollutant and more localized enrichment in Cu, Pb, and As. Magnetic susceptibility, in contrast, is controlled mainly by the concentration of ferrimagnetic particles, especially magnetite-maghemite and Fe-rich technogenic phases produced by brake wear, vehicle abrasion, exhaust residues, and road-surface wear (Gonet et al., 2021; Letaïef et al., 2024). Samples combining increased χ values with elevated pollution indices are likely to reflect a stronger contribution from traffic-derived metal-bearing particles. However, such an arrangement is not expected for all samples, as χ responds mainly to Fe-bearing magnetic carriers, while the geochemical indices respond to PTEs concentration independently of their mineralogical host, which explains the partial mismatch observed in this study. Samples D4 and D6 were the clearest magnetic hotspots, indicating enhanced technogenic ferrimagnetic inputs, although the pollution indices still classified the overall contamination as low to moderate. Conversely, Zn-enriched samples D9 and D17 showed only moderate χ values, suggesting that part of the Zn enrichment was probably associated with weakly magnetic or non-magnetic particles, including tire-wear debris, galvanized material residues, lubricants, or road-surface abrasion products, rather than with strongly ferrimagnetic phases (Councell et al., 2004). The abundant quartz-feldspar matrix may also dilute the magnetic signal, particularly where ferrimagnetic particles occur as a minor component of the dust. Similar behavior was reported in road-dust studies, where the finest fractions may concentrate traffic-related metals and magnetic particles, but the strength of the magnetic response depends on grain size, mineralogy, and the proportion of magnetic carriers (Dytłow et al., 2019). Therefore, magnetic susceptibility should be interpreted as a rapid, source-sensitive indicator of traffic-related ferrimagnetic particles, not as a substitute for geochemical contamination indices, being more informative when used together, as the geochemical indices define contamination intensity and priority elements, while χ supports identifying the contribution of Fe-oxide-rich technogenic particles and refining source interpretation in the school surroundings.

#### Particle-size distribution

**Fig. 4:**
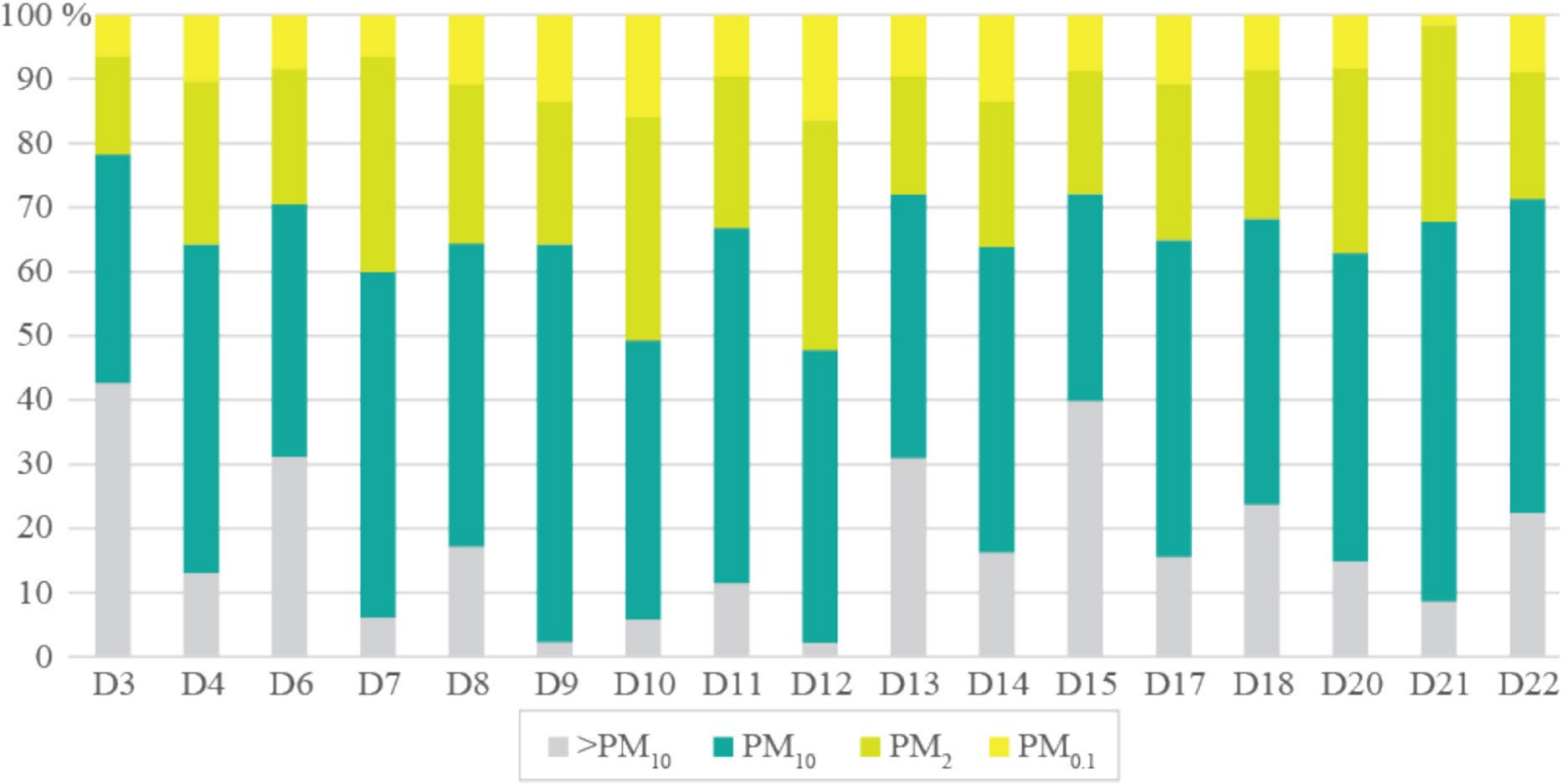
Relative distribution of > PM_10_, PM_10_, PM_2_, and PM_0.1_ mass

The particle-size distribution of the < 106 µm fraction revealed a strong enrichment in fine material across most samples, although with substantial spatial variability (Fig. 4). In this fraction, PM_10_ represented between 47.9 and 95.3% (D22 and D9), while PM_2_ ranged from 19.6 to 71.9% (D3 and D12), and ultrafine PM_0.1_ from 2.5 to 21.1% (D22 and D9). These results suggested that this road dust fraction was dominated by respirable and ingestible particles, consistent with studies reporting strong fining of road-deposited sediments in traffic-affected microenvironments (Amato et al., 2009; Kasimov et al., 2019). Samples D7, D9, D10, D12, and D21 showed particularly high PM_10_ proportions (> 85%), indicating that these locations act as effective traps for fine particulate matter. The elevated PM_10_ content at these sites was consistent with previous findings showing that traffic with frequent braking, acceleration, and idling, especially in school-adjacent streets, enhances emissions of mechanically generated and soot-rich fine particles (Kasimov et al., 2019). Sample D9 also revealed the highest PM_0.1_ proportion (21.1%), reflecting a significant contribution of ultrafine particles, commonly associated with non-exhaust traffic processes, such as brake and tire wear, and thermally generated particles from incomplete combustion, consistent with the street characteristics (curvature, moderate traffic, and speed-variation zones), where braking intensity and turbulent resuspension are higher. Samples D4 and D17, although less extreme, also showed substantial PM_10_ enrichment (78.6 and 74.9%, respectively), values within the range observed in urban roads where fine-particle deposition was favoured by street canyons, reduced wind flushing, and dry deposition during dry periods (Amato et al., 2009). In contrast, D22, which displays the lowest PM_10_ among the enriched samples (47.9%), also presented the lowest PM_0.1_ content (2.5%), suggesting a lower intensity of traffic-related abrasion, likely reflecting fewer braking events, wider road geometry, or enhanced natural ventilation. The pH and EC results of the < 106 µm fraction (Table 2) suggested that samples with the highest PM_10_ and PM_0.1_, e.g., D9, D10 and D12, exhibited low EC, suggesting the dominance of mineral silicate fines (quartz, feldspars, Σphyllosilicates) rather than coarse salt- or cement-derived particles, the presence of soot and carbonaceous particles, and limited retention of soluble salts in the < 2 mm fraction. This trend aligns with the textural behaviour: fine fractions (< 106 µm) generally retain combustion-derived and mechanically abraded particles, while the coarser fraction (< 2 mm) retains salt-rich and carbonate-rich debris, explaining the much higher EC in the coarse fraction. The lowest EC values (D12 21.1 µS/cm; D17 19.9 µS/cm; D22 16.2 µS/cm) corresponded to sites where the fine fraction was dominated by silicate particles with a strong natural lithogenic signal. These samples also showed a relatively reduced PM_0.1_ content, except for D12, where high PM_0.1_ (19.2%) combined with low EC confirmed a composition dominated by traffic-derived ultrafine particles rather than soluble salts. The neutral pH also supported particle stability in the fine fraction. The fine dust fractions are the most likely to become resuspended, adhere to hands and clothes, and contribute to children’s inhalation and ingestion pathways.

#### Individual dust particles morphology and pseudo-total chemical composition

**Fig. 5:**
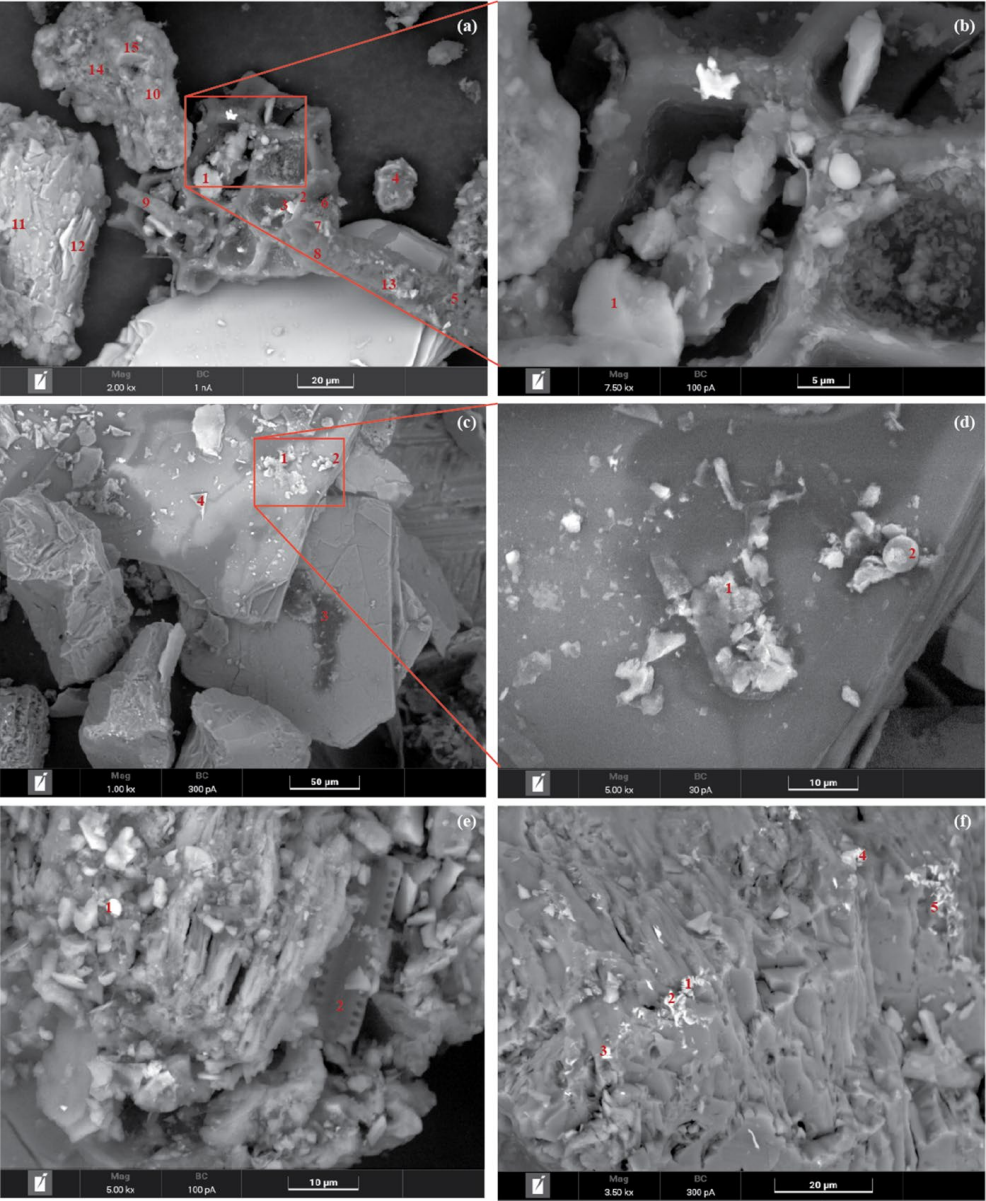
Representative SEM–EDS images of samples D9 (a,b); D10 (c,d); and D22 (e,f)

The SEM–EDS analysis was performed to link particle morphology and composition with the bulk geochemical results, allowing identification of specific carriers of PTEs and discrimination between the geogenic and anthropogenic origin of individual particles in the road dust < 250 µm fraction. Samples D9, D10, and D22 were selected due to the representative particles content. Sample D9 exhibited a heterogeneous mixture of carbonaceous fragments, mineral grains, and metal-rich particles (Fig. 5a; Table S1), consistent with both geogenic and anthropogenic inputs. The SEM–EDS analysis allowed identification of specific particle types acting as carriers of PTEs, supporting bulk geochemical results. Highly carbonaceous particles (points 1–9, 13) are predominant in this sample and can be attributed to carbonaceous material, including anthropogenic components such as asphalt/bitumen binders and soot (black carbon), as well as rubber and plant debris, often with silicate coatings. (Adamiec et al., 2016). A C-rich matrix embedding fine silicate inclusions was identified (point 3), and the larger central particle showed morphology consistent with carbon-rich biochar-like material (honeycomb‑like texture detail in Fig. 5b), potentially derived from combustion processes. These materials, although environmentally beneficial, may pose health risks when inhaled or ingested (Pinelli et al., 2024; Taghlidabad & Sepehr, 2017). Mineral particles are dominated by Si–O with Al and K, corresponding to quartz (point 15), feldspathic/clay minerals partially coated by organic matter (points 10, 11), and clay minerals (points 14) typical of crustal sources (Navarro-Ciurana et al., 2023b). Point 12 composition suggested a clay or Fe-oxide particle with an organic film, all of these phases are consistent with soil-derived material and roadbed aggregates. Metal-rich particles were less abundant but important, such as Fe–Cr-Mn alloy fragments mixed with carbonaceous matter, which showed a typical source related to brake wear or steel abrasion products (Adamiec et al., 2016). Fe-oxide particles (e.g., magnetite-maghemite), identified in bulk XRD, were also observed as angular grains. Sulfur was negligible, while Ti-bearing particles occurred only in trace amounts, likely from pigments or road paint. Additional particle types include carbonaceous spheres with mineral inclusions (combustion residues), soot-rich particles acting as carriers of trace metals, and carbonaceous-silicate aggregates with Fe, Ti, Ca, and K, linked to mixed sources such as traffic emissions, construction dust, and resuspended soil (Gao et al., 2022; Güney & Öz, 2020). Rare particles enriched in rare earth elements (REE; Ce ~ 7 wt%, La ~ 5 wt%) suggested contributions from catalytic converters or industrial emissions (Li et al., 2022). Biological particles (spores, diatoms) were also identified. Sample D9 particle assemblage was consistent with previous studies of urban road dust (e.g., Adamiec et al., 2016; Navarro-Ciurana et al., 2023b). The relatively high organic fraction compared to typical soil-derived dust (> 60% mineral fraction reported by Gunawardana et al. (2012) likely reflects local traffic intensity and asphalt contribution. The presence of fine and ultrafine particles indicates that PTE carriers occur in respirable fractions, consistent with studies showing that PM_10_ hosts most traffic-derived metals and contributes to health risks (Navarro-Ciurana et al., 2023b; WHO, 2021).

Sample D10 contains micron-sized particles with irregular morphologies and assorted chemical compositions (Fig. 5c, d; Table S1), reflecting both geogenic and anthropogenic contributions. The SEM–EDS analysis enabled the identification of particle types acting as carriers of PTEs and supported the interpretation of traffic-related and natural sources. Geogenic mineral particles were represented by aluminosilicate phases, such as point 1, which corresponded to feldspathic or phyllosilicate minerals, such as K-feldspar or micas, with minor Fe, Mg, and K, reflecting accessory phases or substitutions. Similarly, point 4 was consistent with illite, indicating soil-derived material. These particles are typical of local granitoid and metamorphic sources and are commonly found in resuspended soils and construction dust (Candeias et al., 2020; Xie et al., 2005). Anthropogenic carbonaceous particles were also abundant, represented by point 3, identified as carbonaceous material, including soot or other combustion-derived residues, likely from vehicular exhaust. The presence of Fe, S, and Si suggested adsorption of metal-bearing particles or secondary coatings (e.g., magnetite or sulfates). These particles can act as carriers of trace metals and persistent organic pollutants, enhancing their mobility and potential health impact (Tang et al., 2024). Spherical particle in point 2 (Ø ~ 5 µm), composed of Si-Al oxides with high C and K, was interpreted as a combustion-derived aluminosilicate spherule, likely originating from mixed-fuel ash or soot-coated glass (Wilczyńska-Michalik et al., 2020). Additional particle types further indicate traffic-related sources, such as identified metal-rich particles with high Fe (> 40 wt%), Cr (> 9 wt%), Ni (> 5 wt%), and associated Mn, Ti, and Cu, consistent with brake wear and non-exhaust traffic emissions (Grigoratos & Martini, 2015). Cu-rich (> 23 wt%) and Bi-Cu particles (> 29 wt% Bi; > 21 wt% Cu) were also attributed to brake wear, reflecting modern brake pad formulations that use Bi as a substitute for Pb (Grigoratos & Martini, 2015). Particles enriched in REE (Ce > 15 wt%, La > 8 wt%), together with Si, P, and Ag, were indicative of emissions from catalytic converters, brake wear, or industrial sources (Grigoratos & Martini, 2015; Navarro-Ciurana et al., 2023b). Mixed carbonaceous-mineral particles were also observed, including carbonaceous matrices enriched in Fe and S, characteristic of traffic-derived dust, and carbonaceous-silicate aggregates containing Fe, Ti, and aluminosilicates, reflecting resuspension of road dust and combustion residues (Amato et al., 2011; Grigoratos & Martini, 2015). Silicate-oxide particles enriched in K (> 17 wt%) indicated mineral dust mixed with combustion products (Hudson et al., 2004). Additionally, particles containing Na, K, and Cl correspond to deicing salt residues mixed with soot and mineral dust, linked to winter road maintenance (Carvalho et al., 2012).

Sample D22 (Fig. 5e,f; Table S1) showed a clear distinction between metal-rich anthropogenic particles and silicate/carbon particles of predominantly geogenic origin. The SEM–EDS analysis highlighted the presence of traffic-related metallic debris together with natural mineral phases, supporting the interpretation of mixed sources. Metal-rich particles dominated part of the assemblage and were primarily composed of Fe (47–52 wt%), O (15–30 wt%), C (13–22 wt%), with contributions of Cr (3–5 wt%), Cu (1–3 wt%), Sn (0.5–2 wt%), and minor Al, consistent with Fe-oxide fragments and steel/alloy wear particles derived from road-surface abrasion and brake materials, which commonly contain Fe, Cu, Sn, Sb, and carbonaceous binders (Candeias et al., 2020; Denny et al., 2022; Moskovchenko et al., 2022). Point 1 (Fig. 5f), with Fe (42.94 wt%), O (13.10 wt%), Cu (2.64 wt%), and Sn (2.36 wt%), was interpreted as an oxidized Cu-Sn-Fe alloy fragment typical of brake or clutch wear (Miazgowicz et al., 2020). The presence of Cr (up to 8.9 wt%) indicated contributions from stainless-steel or plated components, while elevated carbon contents suggest graphite or organic binders from brake linings. Geogenic and mixed particles were represented by silicate and carbonaceous phases, such as point 3 (Fig. 5f), with high Si (25.53 wt%) and O (38.09 wt%), and minor Al, Na, and K, corresponding to a quartz/feldspar fragment with an organic coating, typical of soil-derived material (Candeias et al., 2020; Moskovchenko et al., 2022). Point 5, characterized by C (21.82 wt%), O (30.80 wt%), and Fe (18.74 wt%), suggested a carbonaceous fragment with embedded Fe, possibly derived from carbonized organic matter or traffic-related debris. Biological particles, including diatom frustules (e.g., genus Nitzschia, sensu lato), were also identified, indicating natural environmental inputs. Additional particle types further illustrated the complexity of sources, with particles showing very high As (> 43 wt%), associated with Fe (> 25 wt%) and S (> 5 wt%), indicative of As-bearing sulfides, likely arsenopyrite-like (FeAsS) fragments or altered sulfide phases. REE-enriched aluminosilicate particles (Ce, La), together with Ba and P, were consistent with emissions from catalytic converters or diesel particulate filter (DPF) ash (Mishra et al., 2022; Vlasov et al., 2022). TiO_2_-rich particles, associated with Ca (> 11 wt%) and Si-Al-O phases, corresponded to road paint materials, where rutile TiO_2_ is used as pigment and CaCO_3_ or BaSO_4_ as fillers (Vlasov et al., 2022). Geogenic mineral fragments were also identified, including Fe-Ti–rich particles (Fe > 35 wt%, Ti > 18 wt%), likely ilmenite (FeTiO_3_), and Zr-rich particles (> 23 wt%), interpreted as zircon (ZrSiO_4_), both typical of crustal sources (Deer et al., 2013). Abundant aluminosilicate particles (feldspars, clays, quartz), sometimes enriched in K, further confirmed contributions from soil and construction materials.

Samples D9, D10, and D22 consistently revealed that urban road dust is a heterogeneous mixture dominated by geogenic mineral particles (quartz, feldspars, and clay minerals) covered with anthropogenic components. In all samples, carbonaceous material, mainly derived from traffic-related sources such as soot, asphalt/bitumen, and combustion residues, was abundant and frequently associated with mineral phases, forming composite particles that can act as carriers of PTEs. Metal-rich particles, including Fe–Cr-Ni-Cu-bearing fragments, Cu- and Bi-rich phases, and alloy particles (e.g., Fe–Cr-Mn and Cu-Sn-Fe), were systematically identified and are consistent with non-exhaust traffic emissions, particularly brake wear, tire abrasion, and mechanical vehicle components (Adamiec et al., 2016; Grigoratos & Martini, 2015; Miazgowicz et al., 2020). The occurrence of REE-enriched particles further supported the contribution from catalytic converters and exhaust-related emissions (Mishra et al., 2022; Navarro-Ciurana et al., 2023b). The identification of Fe-oxide particles (e.g., magnetite-maghemite), together with carbonaceous matrices and fine aggregates, corroborates magnetic susceptibility results and confirms the presence of technogenic magnetic particles associated with traffic activity. Additionally, the presence of < 10 µm metal-bearing and carbonaceous particles across all samples highlighted their relevance for inhalation and ingestion exposure, in agreement with granulometric data showing enrichment in PM fractions.

#### Bioaccessible fractions of the PTEs As, Cr, Cu, Pb, and Ni

**Fig. 6:**
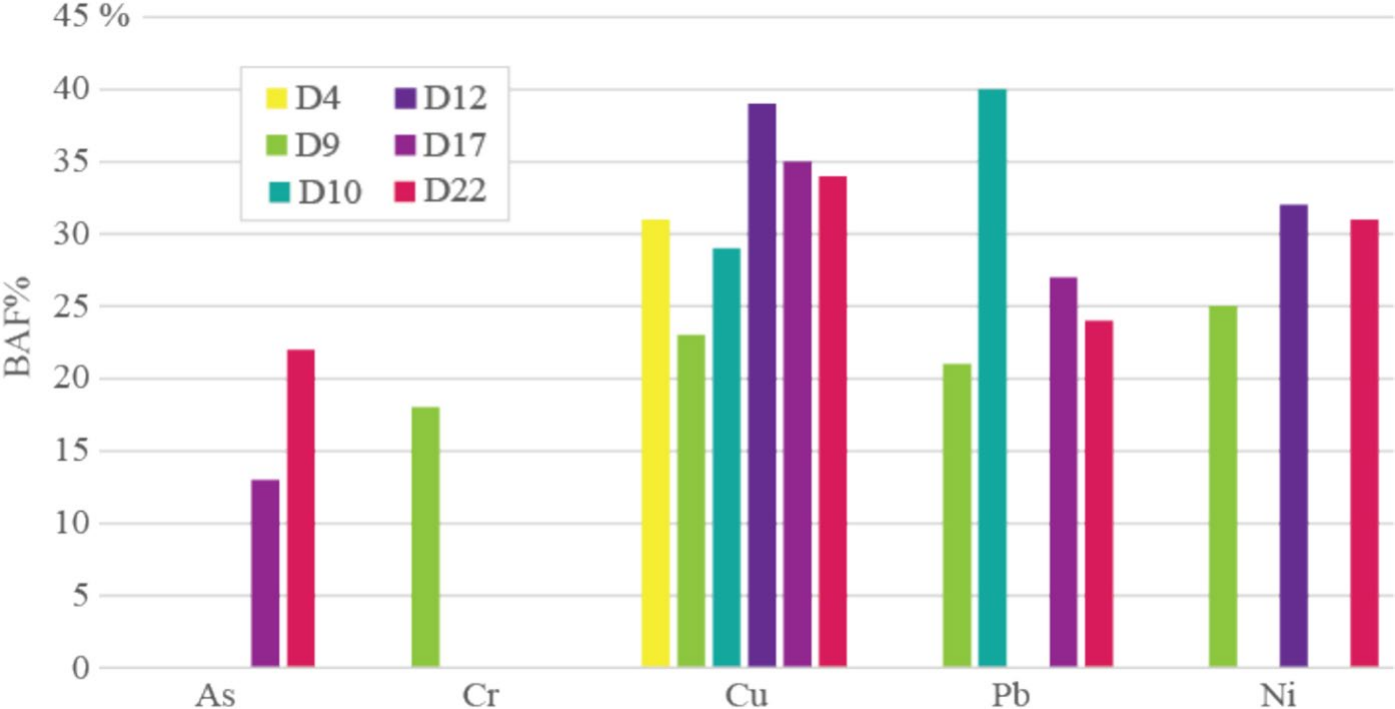
Bioaccessible fractions (BAF%; in %) of As, Cr, Cu, Pb, and Ni, in the < 250 µm fraction of the selected road dust samples D4, D9, D10, D12, D17, and D22

Bioaccessible fractions were evaluated for As, Cr, Cu, Pb, and Ni in the < 250 µm road‐dust samples. D4, D9, D10, D12, D17, and D22 were selected and, submitted to a simulated gastric extraction. These samples were chosen to represent contrasting contamination levels and site characteristics (e.g., traffic intensity, background geology). The results (Fig. 6) showed that As and Cr showed negligible bioaccessibility. However, Cu, Pb, and Ni exhibited significant BAF%. Sample D9 revealed a BAF% of ~ 18% for Cr, ~ 23% for Cu, ~ 21% for Pb, and ~ 25% for Ni, while As revealed a non-existent BAF%. In sample D22, As revealed a BAF% of 22% (sample with the highest total As), and Cu was ~ 34%, Pb ~ 24%, and Ni ~ 31% bioaccessible. Results indicated that bulk As in the road dust was present predominantly in insoluble forms (e.g., Fe-bearing minerals), not dissolving in simulated gastric fluid, while Cu, Pb, and Ni were in more soluble forms. High %BAF suggests that the metal is mobile in acidic gut conditions and poses a higher ingestion risk. For example, Cu and Ni showed BAF% on the order of 23–39%, indicating a large labile pool (higher risk if the dust is ingested). The almost absent As BAF%, except D17 at 13%, implied it is tightly bound, possibly as arsenate strongly adsorbed onto Fe/Al oxides or silicates, consistent with studies showing that Fe/Al-oxides reduce As solubility (Lake et al., 2021). Chromium showed low BAF%, except in D9 (18%), suggesting that a more soluble fraction of Cr, potentially including Cr(VI), was present, since it is more soluble and more mobile (ATSDR, 2016).

At circumneutral pH, many metals precipitate as carbonates or hydroxides, with Pb being reported that in soils with pH > 7.5, carbonates can form stable Pb-carbonate minerals that are insoluble in soil but dissolve in stomach acid, increasing Pb bioaccessibility (Lake et al., 2021). Samples mineralogical composition, with calcite/zeolite, will buffer pH, but will dissolve in acidic conditions. Thus, even if Pb was less available at road dust pH, it will become bioaccessible in vitro. The metalloid As, predominantly present as oxyanion As(V), strongly binds to Fe/Al oxides and clays, and shows little affinity for carbonate phases. As a result, pH variations generally have a limited effect on As mobility. However, increases in pH can enhance As bioaccessibility by reducing the positive surface charge of binding phases and weakening adsorption processes (Lake et al., 2021). In the present samples, which exhibited near-neutral pH and were rich in Fe/Al-bearing minerals, As was therefore expected to remain relatively immobile. Lake et al. (2021) reported that high Fe/Al-oxides content would sequester As and Pb, finding that BAF% decreased significantly with increasing FeOx and linearly with AlOx. The dominant mineral phases in the dust acted to adsorb metals and reduce their solubility in the gut, once As showed essentially no BAF%, reflecting strong binding, while Cu and Ni, which often occur in more labile carbonates or silicate structures, showed high dissolution. The EC revealed soluble salts, with sample D9 showing high EC and Cu/Ni BAF%, suggesting that dust with more soluble salts and low pH tended to have slightly higher BAF%, consistent with the chemical composition, since metals dissolve more as pH decreases. Road dust near-neutral pH, and with Fe/Al-rich minerals, suppressed As bioaccessibility, but a significant mobile fraction of Cu, Ni, and Pb was retained, which is an important factor for ingestion risk.

The selected road dust total concentration, on the < 250 µm fraction, exceeded health-based soil/dust guidelines, e.g., D22 As and Cu content revealed a higher content than the CCME (2025) human-health soil guideline of 12 and 63 mg/kg, respectively (for all land uses). Also, sample D12 presented higher Ni and Pb than the 45 mg/kg (CCME, 2025) and 200 mg/kg (USEPA, 2024) guidelines for residential/environmental. However, these guidelines assume high bioavailability (~ 100% absorption), and the studied samples BAF% revealed that only a fraction of the total chemical composition was actually bioaccessible. For As, the effective hazard was lower than the total content suggested, since only a fraction of the total concentration is bioaccessible in gastric conditions (children’s exposure via dust ingestion would be proportionally smaller) (Lake et al., 2021). In contrast, Ni and Cu with higher BAF% content suggested that a larger fraction of the total dose was available. When comparing to health criteria, some pseudototal values exceeded safe soil levels (WHO/CCME/USEPA), and the bioaccessible portion for Ni, Cu, and Pb was enough to justify concern, e.g., specifically for Pb, a guide value of 200 mg/kg soil screening, below which residential risk is deemed low (USEPA, 2024).

Children are especially at risk from dust ingestion due to hand-to-mouth behavior. It is well documented that toddlers ingest tens to hundreds of milligrams of dust/soil each day (ATSDR, 2018). Therefore, even trace bioaccessible fractions can be of concern due to cumulative dose, e.g., if a child ingests 100 mg/day of D9 road dust, the absorbed Cu dose would be ~ 0.024 mg Cu/day, and Ni ~ 0.016 mg Ni/day. Over time, this could contribute significantly to total intake, potentially exceeding safe reference doses, especially when combined with dietary sources. Given that some BAF% some values exceeded 30%, the health risk from Ni and Cu in the studied road dust cannot be considered 100% without risk. By contrast, As low bioaccessibility fraction reduces its hazard from incidental ingestion, illustrating why in situ mobility (not just total content) can be considered critical for risk. The combination of high total concentrations and substantial bioaccessible fractions of Ni, Cu, and Pb suggested a significant risk to children from hand‐to‐mouth exposure, whereas As, despite its high total concentration, largely immobile under gastric conditions.

#### Road dust composition: potential health impacts

The health potential effects of some PTEs in road dust are variable, depending on the element, content, and exposure route. Arsenic, classified as a human carcinogen (IARC, 2025a; Group 1), both by ingestion and inhalation, occurs in road dust predominantly in inorganic forms, mainly as arsenate (As(V); e.g., H_2_AsO_4_^−^, HAsO_4_^2−^) and arsenite (As(III); e.g., H_3_AsO_3_) (ATSDR, 2021b). Chronic inhalation of As-enriched dust can cause lung cancer, as well as skin, bladder, and liver cancers in exposed populations, and also produces acute respiratory irritation and systemic symptoms (e.g., nausea, diarrhea) (ATSDR, 2021b). Dermal contact with inorganic As causes skin lesions and pigmentation changes (often a precursor to skin cancer), while ingestion of As (e.g., contaminated soil or crops) causes gastrointestinal distress acutely, and, over the years, leads to keratoses, hyperpigmentation, and multiple internal cancers. On a molecular level, arsenic disrupts signal transduction and causes gene silencing via oxidative damage and methylation interference (ATSDR, 2021b).

The toxicity of Cr depends on its oxidation state, once trivalent Cr(III) is an essential micronutrient at trace levels, but hexavalent Cr(VI), used in chrome plating and pigment, is highly toxic (readily penetrates cells and is reduced to Cr(III), generating reactive intermediates that cause double-strand DNA breaks) (NTP, 2021). Industrial exposure to Cr(VI) is epidemiologically linked to lung cancer and nasal cancer, and chronic exposure causes bronchitis and fibrosis (NTP, 2021), with IARC (2025a) classifying Cr(VI) compounds as a human carcinogen (Type 1). Even though Cr(III) is less harmful, fine particles can carry oxidized forms, and mechanistically, Cr induces inflammation, oxidative stress, and chromosomal mutations in lung tissue (sister chromatid exchanges, micronuclei) (NTP, 2021).

Nickel compounds are also classified as human carcinogens (IARC, 2025a; Group 1), occurring in road dust from, e.g., stainless steel wear, fuel additives, and industrial emissions. Inhalation of Ni-containing dust, especially Ni-oxide or sulfide, causes chronic bronchitis and is strongly linked to lung and sinonasal cancers (IARC, 2025a). Apart from cancer risk, Ni causes allergic contact dermatitis, which is the most common health effect in the general population, and also can induce oxidative DNA damage and alter immune responses (ATSDR, 2021a). Copper is an essential nutrient, but at high levels can be toxic, and in road dust can be from brake pads, brake lining wear, and plumbing corrosion. Reports showed that chronic exposure induced respiratory symptoms and decreased lung function, but Cu is generally less harmful by inhalation than Ni or Cr (NIOSH, 1994). Ingestion of excessive Cu can cause gastrointestinal distress (vomiting, diarrhea) and, in extreme cases (e.g., Wilson’s disease), liver toxicity (NIOSH, 1994). Mechanistically, copper participates in redox cycling (Cu⁺/Cu^2+^) and can catalyze the formation of free radicals; however, the human body tightly regulates Cu. Road dust Cu is mostly a concern for acute irritation rather than a potent carcinogen.

Cadmium is a highly toxic metal found in road dust from tire wear, brake linings, and fossil fuel residue, also classified as human carcinogenic (IARC, 2025a; Group 1). Systemically, inhaled or ingested Cd accumulates in the kidney, binding metallothionein and being retained in proximal tubules, overtime this causes Cd nephropathy, a tubular dysfunction, and eventual kidney failure (ATSDR, 2015). Additionally, Cd also displaces Ca, leading to bone demineralization and fractures (“Itai-itai disease” features), long-term, low-level Cd exposure causes urinary protein loss, and if high enough, kidney disease, and there is also evidence that cadmium exposure increases the risk of prostate and kidney cancer, though lung cancer is most prominent (ATSDR, 2015) Zirconium occurs in road dust mainly from abrasive materials, e.g., powdered Zr in brake pads, and as a trace element in concrete road surfaces, but is not considered a systemic toxicant at environmental levels, while inhaled Zr-containing dust can cause lung pathology (NIOSH, 2011). Zr is poorly absorbed and largely inert, with dermal exposure occasionally causing contact granulomas on the skin. No strong evidence links Zr to cancer or systemic toxicity, with the main health effect in road dust scenarios being potential chronic irritation and granuloma formation in the lungs of heavy, long-term exposure.

The REE Ce and La, used in catalytic converters, spark plugs, and high-tech devices, are increasingly found in road dust near industrial zones and heavy traffic (Navarro-Ciurana et al., 2023a). Rare earths have low acute toxicity, but emerging evidence suggests subtle health effects. Studies have found elevated Ce and La in the blood and hair of residents near e‑waste sites and high-traffic areas, and that hormonal and cardiovascular associations, for example, higher Ce/La in serum, were linked to altered thyroid-stimulating hormone (TSH) levels (Brouziotis et al., 2022). In women undergoing IVF, higher La in serum correlated with worse pregnancy outcomes (more spontaneous abortions) (Brouziotis et al., 2022). Cerium levels in toenails have been associated with an increased risk of acute myocardial infarction, and Ce and La may act as endocrine disruptors or affect vascular health at high exposures (Brouziotis et al., 2022). Despite road dust studies often finding Ce/La enrichment, relative to background soil, the overall cancer risk from these REEs in urban dust appears very low (Brouziotis et al., 2022). Nevertheless, their potential for bioaccumulation and neurological effects (e.g., deposition in brain tissues of exposed animals) warrants concern, especially in occupational settings.

Even when the PM_10_ fraction of road dust is small, it can carry a disproportionate share of heavy metals. For example, the ultrafine (PM_0.1_) fraction of resuspended road dust, only ~ 2% of the PM_10_ mass, was found to contain 2–3 times higher concentrations of Cd, Cr, Zn, and V than the bulk dust (Beauchemin et al., 2021). Particles in the PM_2.5–10_ range showed enrichment of Cu, Zn, Sb, and Ti relative to larger grains (Beauchemin et al., 2021). Road dust metals tend to adhere to or generate nanoscale particles, especially hazardous because fine particles penetrate deeply into the lung, with PM_1_ reaching the alveoli where they can release metals directly into lung tissue or blood (Beauchemin et al., 2021). The ultrafine particles carrying a heavy-metal payload may be more toxic than a larger particle of equal mass, due to their greater surface area and bioavailability. Beauchemin et al. (2021) reported that as road dust particles size decreased, the concentration of PTEs (Zn, Cu, Cr, Pb, Cd) increased significantly, and that even though pavement dust contributes with a smaller fraction to airborne PM_10_, its molten-metal-nanoparticle component (from brakes) is especially concentrated in metals. Even if PM_10_ reveals low values, this small fraction of airborne dust from road surfaces can contain very high metal doses, raising a special concern for vulnerable groups, such as children breathing road dust or playing on streets that may inhale a lung-toxic dose of Cd, Cr, or Ni without it registering as high PM_10_ mass. Furthermore, fine particles have longer atmospheric residence times and wider dispersal, with windborne road dust nanoparticles that can travel beyond traffic corridors, exposing nearby residents and contributing to urban airborne toxic load. Regulatory PM standards focus on mass (PM_10_, PM_2.5_), but these do not capture the toxic potential of trace-metal-rich fines. Road dust small fine fraction can carry an unseen dose of PTEs, even if PM_10_ levels are low, the fine tail of the dust size distribution can deliver outsized heavy-metal exposures (Beauchemin et al., 2021).

Preventive measures (street cleaning, controlling non-exhaust emissions, and reducing traffic on dusty roads) can reduce the total PTEs load. From a public health perspective, even trace metals in road dust deserve attention because of these mechanistic hazards. Inhalation of fine metals in dust is particularly dangerous, as particles may deposit deep in the lung and release PTEs at the sensitive respiratory tissue, or even translocate systemically (Wang et al., 2024). Dermatological exposure is generally minor except for Ni allergy, but ingestion (especially in children) can lead to direct gastrointestinal absorption of metals that accumulate in organs (ATSDR, 2021a). The present study of road dust revealed the presence of PTEs (e.g., As, Cr, Cu, Ni, Cd, and emerging elements Ce, La, Zr) that can harm human health through multiple pathways. Even when fine dust is scarce, particles can transport significant metal loads into the body. Public health efforts should account for these hazards by monitoring road dust PTEs, reducing sources, and mitigating exposures, which can help limit this “invisible” exposure to airborne heavy metals (Wang et al., 2024).

From a management and policy perspective, the results indicate that broad‐scale controls may not be required, but targeted interventions at the hotspot sites are justified. Actions such as more frequent sweeping of road dust, reducing resuspension (via wet cleaning or vegetation buffer strips), controlling brake/tire material emissions, and regular monitoring of dust fractions (< 250 µm, < 10 µm) would help reduce potential human‐exposure pathways. Future monitoring should include periodic sampling to detect upward trends, especially as traffic volumes or vehicle types change. Also, if human-health risk assessment is pursued, the elevated Zn hotspots may warrant speciation/bioavailability analysis since fine dust-bound metals may have enhanced mobility and exposure potential.

### Indoor Rn in the public schools

**Fig. 7:**
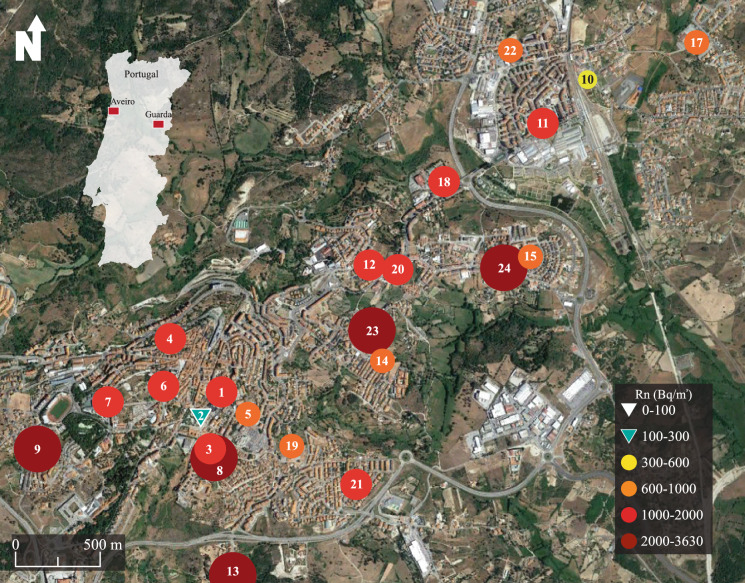
Radon concentration in the 23 monitored schools (in Bq/m^3^)

In the 23 public schools monitored, extremely high indoor Rn concentration was found, with an average of 1540 ± 921 Bq/m^3^, a value over five times the Portuguese legal reference level of 300 Bq/m^3^ (DR 232/2018 2018) for indoor air. Radon levels varied among the surveyed schools, ranging from 133 Bq/m^3^ to a maximum of 3630 Bq/m^3^ in one nursery school (Fig. 7). Remarkably, only one of the 23 schools monitored showed indoor Rn concentration below the 300 Bq/m^3^, and none below the WHO (2023) recommended level of 100 Bq/m^3^. This guideline was primarily established for residential environments, yet it is widely applied as a general benchmark for indoor environments, including schools, representing a conservative public health reference for indoor Rn exposure in different environments, both for children and adults. The vast majority greatly exceeded both limits, with five schools (22% of the sample) showing Rn concentration > 2000 Bq/m^3^, ten (43%) between 1000 and 2000 Bq/m^3^, six (26%) between 600 and 1000 Bq/m^3^, and one (4%) between 300 and 600 Bq/m^3^, confirming a preliminary Rn survey between March to May, 2019, in the same schools (Candeias et al., 2021), and being levels above global indoor Rn averages, usually 50–100 Bq/m^3^. This contrast is also evident when compared with Aveiro public schools, where an average concentration of 99 ± 35 Bq/m^3^ was measured, a location initially considered as a control area due to its predominantly sedimentary geological setting (e.g., coastal sands, silts, and clay-rich deposits). These values are close to the WHO (2023) suggested reference value, indicating that even in areas with lower Rn potential, indoor concentrations may approach guideline values due to building characteristics and ventilation conditions, still markedly lower than those observed in the schools of the granitic Rn-prone region of Guarda.

Several factors probable contributed to such high values in Guarda public schools, such as, the geological setting, being a region on granitic bedrock, known to emit abundant Rn gas (Domingos et al., 2021; Louro et al., 2013); and the winter conditions and building ventilation practices with classroom windows generally closed due to the cold, limiting air exchange, creating an environment suitable for Rn concentration. It is important to note that the reported measurements were conducted during the winter, representing a worst-case scenario. This is supported by Louro et al. (2013), who observed seasonal variations in Rn concentrations between winter and summer in the study area, with the highest levels occurring during the cold period. The Rn concentration in northern European countries was generally about 50% higher than that in southern Europe, primarily due to the colder climate (Arvela, 2005). According to this author, in houses where soil Rn was the dominant source, summer minimum concentration was typically around 50% of the winter maximum. Assuming this seasonal contrast, the estimated annual average Rn concentration would be approximately 1155 Bq/ m^3^, which is nearly four times the Portuguese legal reference level (DR 232/2018 2018). A classroom with ~ 1500 Bq/m^3^ Rn corresponds to 15 times the WHO (2023) reference level, comparable to working in a U mine with no ventilation, in terms of Rn exposure, confirming a significant public health issue for the region, pointing to an urgent need for remediation and further investigation. Simple measures such as improving ventilation can benefit indoor air quality, but levels in the thousands of Bq/m^3^ likely require more intensive mitigation.

The health risk of the measured high Rn concentrations in the public schools was calculated (USEPA, 2025) and showed that Total Risk over a lifetime ranged from 2.03E-06 to 8.53E-06, with schools presenting a risk between 1.00E-06 and 1.00E-05, corresponding to a risk range where mitigation measures are recommended, even though values were below the upper threshold of 1.00E-04 (unacceptable risk) (Table 6). Only one school exhibited a Total Risk below 1.00E-06 (D2 6.19E-07), being classified as negligible risk. Results confirmed that, from a regulatory perspective, the dominant concern is risk management rather than emergency-level intervention (USEPA, 2023, 2025). Total Risk reflects the inhalation exposure under realistic indoor residence conditions, representative of school environments. The Total Risk, equivalent to the inhalation ambient air risk, as Rn exposure in indoor environments occurs almost exclusively through inhalation, a pathway dominance consistent with the established radiological protection principles, which recognizes Rn progeny inhalation as the primary mechanism leading to α-particle irradiation of bronchial epithelium and subsequent lung cancer risk (IARC, 2025b; UNSCEAR, 2021). The Total Risk (no decay) ranged from 1.02E-03 to 3.41E-02, among all schools, corresponding to the Inhalation Risk (no decay), with the “no decay” representing a highly conservative modeling assumption in which radioactive decay and progeny loss are not accounted for during exposure and transport. The calculations intentionally overestimate the dose and risk, providing an upper-bound screening estimation, rather than a realistic exposure outcome (USEPA, 2025), while Total Risk incorporates decay and equilibrium considerations, more representative of real indoor conditions. Therefore, the high Total Risk (no decay) values should be interpreted as the worst-case scenario, reinforcing the importance of mitigation, but not as realistic predictions of lifetime cancer risk. From a public health perspective, the Total Risk results were particularly relevant for children, who represent a sensitive population due to their higher respiration rates relative to body mass, ongoing lung development, and longer post-exposure lifetime expectancy. Epidemiological studies have shown that Rn exposure contributes to lung cancer risk even at concentrations below regulatory limits, with a near-linear dose–response relationship and no evidence of a safe threshold (Darby et al., 2005; WHO, 2009). Exposure during childhood adds cumulatively to lifetime risk, making school environments critical targets for prevention strategies (Kendall & Smith, 2002; UNSCEAR, 2021). Guarda schools represent an example of chronic low-to-moderate cancer risk, distributed across a large number of children over many years, which can translate into a non-negligible population-level health burden. These findings support the urgent implementation of Rn mitigation measures, including improved ventilation, structural remediation, and systematic post-mitigation monitoring, particularly in educational facilities located in granitic regions.

**Table 6:** Lifetime excess cancer risk associated with indoor Rn exposure in the 23 public schools monitored, being risk thresholds Risk < 1.00E-06, no significant risk, values in underlin; 1.00E-06 < Risk < 1.00E-04, mitigation recommended, values in italic; and Risk > 1.00E-04, high and unacceptable risk, values in bold

ID	Total risk	Total risk (no decay)	ID	Total risk	Total risk (no decay)
D1	*4.96E-06*	**8.20E-03**	D13	*2.09E-05*	**3.40E-02**
D2	6.19E-07	**1.02E-03**	D14	*4.59E-06*	**7.60E-03**
D3	*9.82E-06*	**1.61E-02**	D15	*4.97E-06*	**8.21E-03**
D4	*5.51E-06*	**9.11E-03**	D17	*5.58E-06*	**9.23E-03**
D5	*4.24E-06*	**7.01E-03**	D18	*1.11E-05*	**1.81E-02**
D6	*7.18E-06*	**1.18E-02**	D19	*3.70E-06*	**6.11E-03**
D7	*1.14E-05*	**1.87E-02**	D20	*9.23E-06*	**1.52E-02**
D8	*5.75E-06*	**9.51E-03**	D21	*9.12E-06*	**1.50E-02**
D9	*9.40E-06*	**1.54E-02**	D22	*5.01E-06*	**8.29E-03**
D10	*2.03E-06*	**3.35E-03**	D23	*1.89E-05*	**3.08E-02**
D11	*1.08E-05*	**1.78E-02**	D24	*2.10E-05*	**3.41E-02**
D12	*1.04E-05*	**1.70E-02**			

The integrated assessment combining outdoor road dust characterization with indoor Rn monitoring provided a more complete exposure framework, linking particulate-bound contaminants with inhalation of a radioactive gas that accumulates indoors. This dual approach captures both source-to-sink pathways (traffic-derived dust infiltration and resuspension) and indoor accumulation processes (Rn entry, retention, and variability), allowing a more realistic estimation of cumulative exposure, particularly for sensitive populations such as children. This integrated perspective aligns with recommendations emphasizing multi-exposure pathway assessments in indoor environments, as highlighted by WHO (2009), which underscores the need to consider combined environmental exposures when evaluating health risks.

## Conclusions

This study integrates outdoor road dust and indoor Rn to provide a coherent view of environmental exposure in schools in Guarda. Road dust reflects a mixed geogenic-anthropogenic system, dominated by quartz-feldspar assemblages derived from local geology, but clearly modified by traffic activity, construction materials, and winter road maintenance. Physicochemical conditions (near-neutral to slightly alkaline pH, high EC, variable OM) supported this interaction, influencing the retention and behavior of potentially toxic elements. The PTEs showed overall low to moderate contamination, but with clear spatial variability and localized hotspots, particularly for Zn and, to a lesser extent, As. Contamination indices confirmed this pattern, identifying Zn as the dominant pollutant and indicating that contamination is not uniform but controlled by traffic intensity, braking zones, and dust accumulation near specific schools. Magnetic susceptibility reinforced this interpretation, pointing to the presence of traffic-derived ferrimagnetic particles and supporting its use as a rapid proxy for anthropogenic inputs. The strong enrichment in fine fractions (PM_10_, PM_2_, PM_0.1_) was a key aspect of exposure, since these fractions concentrate carbonaceous material and metal-bearing particles, including brake-wear debris, alloy fragments, soot, and catalyst-related phases identified by SEM–EDS. Their mobility and accessibility make them the main pathway for inhalation and ingestion, particularly for children. This is further constrained by bioaccessibility results, which showed that while As and Cr are largely immobile, Cu, Pb, and Ni exhibited significant soluble fractions, indicating that exposure risk was governed more by chemical availability than by total concentration. In contrast, indoor Rn represents a more critical and pervasive exposure pathway, with most schools exceeding the 300 Bq/m^3^ reference level, and all exceed the more conservative 100 Bq/m^3^ threshold, with concentrations driven by the granitic geological setting and limited ventilation. The associated risk values consistently fall within the range where mitigation is recommended, highlighting Rn as a priority issue in these environments. Results pointed to a combined exposure scenario, where fine, metal-bearing dust and elevated indoor Rn coexist and affect the same sensitive population. This reinforces the need for integrated assessment, as outdoor particulate sources and indoor radiological exposure cannot be considered independently in school environments. Future work will prioritize systematic and seasonal monitoring of both dust and Rn, with repeated sampling at identified hotspots and greater focus on the finest inhalable fractions. The inclusion of lung bioaccessibility assays is necessary to better constrain inhalation risk. Mitigation should be targeted and site-specific, combining measures to reduce dust accumulation and resuspension near schools, control non-exhaust traffic emissions, and implement effective Rn reduction strategies, including improved ventilation, sealing of entry pathways, and structural remediation where required, all supported by continuous post-mitigation monitoring.

## Supplementary Information

Below is the link to the electronic supplementary material.

Supplementary file1 (PDF 231 KB)

## Data Availability

The data underlying this article will be shared on reasonable request to the corresponding author.
