# Supplementary material for: Multi-hazard exposure in public schools of a medium-sized city: outdoor road dust contamination, and indoor Rn risk
Source: Environ Geochem Health. 2026 Jun 29;48(10):423. doi: 10.1007/s10653-026-03313-6 (PMC13315333; doi:10.1007/s10653-026-03313-6)
Supplement: Supplementary file 1 — Supplementary file1 (PDF 231 KB) [file 10653_2026_3313_MOESM1_ESM.pdf]

Multi-hazard exposure in public schools of a medium-sized city: outdoor road dust contamination, and indoor Rn risk

Lara Almeida[1], Fernando Rocha[1], Alcides Pereira[2], Cristina Sequeira[1], Carla Candeias[1*]

[1]GeoBioTec Research Unit, Department of Geosciences, University of Aveiro. Campus de Santiago, 3810-193 Aveiro, Portugal

[2]University of Coimbra, Institute D. Luiz, Department of Earth Sciences, Coimbra, 3030-299, Portugal

*Corresponding author (candeias@ua.pt)

Table S1. Representative identified particles, classification and interpretation for sample D9, D10, and D22.

Particle type	Main composition	Interpretation	Probable source	References
		**Sample D9**		
Carbonaceous particles	C (50-78 wt.%), O, trace Si/Al/K	Organic matter (soot, asphalt, rubber, plant debris)	Traffic emissions, asphalt, urban OM	Adamiec et al. 2016
C-rich matrix with silicates	C, O, Si+Al	Carbon matrix with mineral inclusions	Mixed anthropogenic/geogenic	Adamiec et al. 2016
Biochar-like particle	C-rich	Combustion-derived carbon material	Biomass/combustion residue	Hamzenejad Taghlidabad & Sepehr 2017; Pinelli et al. 2024
Quartz	Si-O + surface C	Soil mineral	Geogenic	Navarro-Ciurana et al. 2023b
Feldspar/clay particles	Si-Al-K-O	Aluminosilicates	Soil/construction dust	Navarro-Ciurana et al. 2023b
Clay/Fe-oxide with OM	Al-Si-Fe-Mg-O + C	Mineral with organic coating	Mixed geogenic/anthropogenic	-
Metal alloy particle	Fe-Cr-Mn + C	Brake/steel abrasion	Traffic (brake wear)	Adamiec et al. 2016
Fe-oxide particles	Fe-rich	Magnetite-maghemite	Traffic/combustion	-
Carbonaceous spheres	C + inclusions	Combustion residue	Vehicle exhaust, asphalt	Güney & Öz 2020
REE-rich particles	Ce, La	Catalyst-related	Vehicle catalysts/industry	Li et al. 2022
Carbon-silicate aggregates	C + Si + Fe/Ti/Ca/K	Mixed aggregates	Traffic + soil + construction	Gao et al. 2022
Ti-bearing particles	Ti traces	Pigments/paint	Road marking/urban materials	Gao et al. 2022
Biological particles	-	Diatoms, spores	Natural input	-
		**Sample D10**		
Aluminosilicates (feldspar/mica)	Si-Al-O (+K, Fe, Mg)	Feldspathic/Σphyllosilicates minerals	Geogenic (granitoids, soils)	Candeias et al. 2020
Illite (clay mineral)	Si-Al-K-O	Clay mineral	Soil-derived	Xie et al. 2005
Carbonaceous particles (soot)	C-rich + O (+Fe, S, Si)	Combustion-derived carbonaceous material	Vehicle exhaust	Tang et al. 2024
Aluminosilicate spherules	Si-Al-O + C, K	Combustion-derived ash/glass particles	Mixed-fuel combustion	Wilczyńska-Michalik et al. 2020
Metal-rich particles	Fe-Cr-Ni-Mn-Ti-Cu	Alloy/oxide fragments	Brake wear, traffic	Grigoratos & Martini 2015
Cu-rich particles	Cu (>23 wt.%)	Metallic particles	Brake wear	Grigoratos & Martini 2015
Bi-Cu particles	Bi (>29 wt.%), Cu (>21 wt.%)	Brake pad material	Traffic (modern brakes)	Grigoratos & Martini 2015
REE-rich particles	Ce, La, Si, P, Ag	Catalyst-related particles	Catalytic converters/industry	Navarro-Ciurana et al. 2023b
Carbonaceous-Fe-S particles	C + Fe + S	Traffic-related aggregates	Exhaust + wear	Grigoratos & Martini 2015
Carbonaceous-silicate aggregates	C + Si + Fe/Ti	Mixed particles	Resuspension + combustion	Amato et al. 2011
K-rich silicate particles	Si-O + K (>17 wt.%)	Mineral dust + combustion	Mixed geogenic/anthropogenic	Hudson et al. 2004
Salt-bearing particles	Na-K-Cl	Deicing residues	Road maintenance	Carvalho et al. 2012
		**Sample D22**		
Fe-rich metal particles	Fe-O-C + Cr, Cu, Sn	Fe-oxide/alloy fragments	Brake wear, steel abrasion	Candeias et al. 2020; Denny et al. 2022; Moskovchenko et al. 2022
Cu-Sn-Fe alloy fragment	Fe, Cu, Sn	Oxidized alloy particle	Brake/clutch wear	Miazgowicz et al. 2020
Stainless-steel particles	Fe-Cr	Alloy fragments	Traffic/metal wear	-
Carbonaceous metal particles	C + Fe/Cr/Cu	Graphite/binder-rich particles	Brake linings	-
Quartz/feldspar fragment	Si-O (+Al, Na, K)	Silicate mineral	Soil-derived	Candeias et al. 2020; Moskovchenko et al. 2022
Carbonaceous Fe-bearing fragment	C-O-Fe	Carbonaceous debris with metals	Traffic/organic residue	-
As-bearing sulfide	As-Fe-S	Arsenopyrite-like particle	Geogenic/industrial	-
REE-rich particles	Ce, La, Ba, P	Catalyst/DPF-related	Traffic emissions	Mishra et al. 2022; Vlasov et al. 2022
TiO_2-rich particles	Ti-Ca-Si-Al	Pigment + fillers	Road paint	Vlasov et al. 2022
Ilmenite	Fe-Ti	Fe-Ti oxide mineral	Geogenic	Deer et al. 2013
Zircon	Zr-rich	Zr silicate mineral	Geogenic	Deer et al. 2013
Aluminosilicates	Si-Al-K-O	Feldspars/clays	Soil/construction dust	-
Biological particles	-	Diatom (Nitzschia)	Natural input	-
